# Bacterial Human Virulence Genes across Diverse Habitats As Assessed by *In silico* Analysis of Environmental Metagenomes

**DOI:** 10.3389/fmicb.2016.01712

**Published:** 2016-11-03

**Authors:** Ditte A. Søborg, Niels B. Hendriksen, Mogens Kilian, Jan H. Christensen, Niels Kroer

**Affiliations:** ^1^Department of Environmental Science, Aarhus UniversityRoskilde, Denmark; ^2^Research Group for Energy and Environment, VIA University CollegeHorsens, Denmark; ^3^Department of Biomedicine, Aarhus UniversityAarhus, Denmark; ^4^Department of Plant and Environmental Sciences, University of CopenhagenFrederiksberg, Denmark; ^5^Department of Biology, University of CopenhagenCopenhagen, Denmark

**Keywords:** bacteria, environmental metagenomes, evolution, *in silico*, non-host, virulence

## Abstract

The occurrence and distribution of clinically relevant bacterial virulence genes across natural (non-human) environments is not well understood. We aimed to investigate the occurrence of homologs to bacterial human virulence genes in a variety of ecological niches to better understand the role of natural environments in the evolution of bacterial virulence. Twenty four bacterial virulence genes were analyzed in 46 diverse environmental metagenomic datasets, representing various soils, seawater, freshwater, marine sediments, hot springs, the deep-sea, hypersaline mats, microbialites, gutless worms and glacial ice. Homologs to 16 bacterial human virulence genes, involved in urinary tract infections, gastrointestinal diseases, skin diseases, and wound and systemic infections, showed global ubiquity. A principal component analysis did not demonstrate clear trends across the metagenomes with respect to occurrence and frequency of observed gene homologs. Full-length (>95%) homologs of several virulence genes were identified, and translated sequences of the environmental and clinical genes were up to 50–100% identical. Furthermore, phylogenetic analyses indicated deep branching positions of some of the environmental gene homologs, suggesting that they represent ancient lineages in the phylogeny of the clinical genes. Fifteen virulence gene homologs were detected in metatranscriptomes, providing evidence of environmental expression. The ubiquitous presence and transcription of the virulence gene homologs in non-human environments point to an important ecological role of the genes for the activity and survival of environmental bacteria. Furthermore, the high degree of sequence conservation between several of the environmental and clinical genes suggests common ancestral origins.

## Introduction

Bacterial pathogens continue to cause problems for humans by the continuous evolution of known pathogens and emergence of new pathogens (Jackson et al., 2011). Approximately 160 new bacterial infectious diseases have been discovered between 1940 and 2010, and emerging infectious diseases constitute a significant burden on global economies and public health (Jones et al., 2008). While the mechanisms by which many pathogenic bacteria cause disease are relatively well-described, the evolutionary origin of virulence is not fully understood (Wilson et al., 2002; Persson et al., 2009). Bacterial virulence determinants causing disease in humans may evolve through complex host-pathogen interactions (Wilson and Salyers, 2003; Mikonranta et al., 2012) but their origin may also lie in non-human reservoirs amongst the environmental microbiota (Martínez, 2012).

The large majority of studies on virulence in the environment have focused on the detection and survival of clinically relevant bacteria in non-host environments (Garcia-Aljaro et al., 2009; Vezzulli et al., 2010; Davidson et al., 2015). Only few studies (e.g., Persson et al., 2009; Søborg et al., 2013) have targeted the environmental gene pool of pathogenic traits, hence evidence of the presence of virulence genes in environmental microbiomes is sparse.

By PCR and cultivation-based approaches we have previously investigated the presence of virulence factors in soil and freshwater environments (Søborg et al., 2013, 2014). However, to better understand (i) the significance of virulence gene homologs for bacterial survival in non-human environments and (ii) the role these environments play for the evolution of bacterial human pathogens, a much broader investigation across multiple environmental microbiomes is required. The increasing availability of environmental metagenomes provides an ideal opportunity for interrogating the occurrence of bacterial human virulence genes in natural environments. Hence, we performed an *in silico* analysis targeting 24 bacterial human virulence genes in 46 environmental bacterial metagenomes. The analysis showed that not only were the bacterial virulence gene homologs widespread in natural (non-human) environments, evidence of environmental transcription was also observed as was a high degree of sequence conservation between some of the environmental and clinical genes. Thus, the results provide documentation of an important role of virulence gene homologs in environmental microbiomes and suggest they may be precursors of virulence genes seen in clinically relevant pathogenic bacteria.

## Materials and methods

### Blast searches for virulence determinants in metagenomic datasets

Complete translated sequences of 24 bacterial virulence genes were used as queries in TBLASTN searches in 46 environmental metagenomes of the Community Cyberinfrastructure for Advanced Microbial Ecology Research and Analysis (CAMERA) database (Sun et al., 2011; note that the database has been transferred to iMicrobe (http://data.imicrobe.us). The genes covered several classes of virulence determinants including toxin genes (*hlyA, hylA, ply, stx1, stx2, vacA, hlgB*), adhesin genes (*papA, papH, papC, fimH, focG*), secretion genes (*invA, spiA, sipB, sipC, ssaD*), inflammatory genes (*wbdI, rfbE*), regulatory genes (*phoP, phoQ*) and resistance genes (*pqaB, mecA, yfbI*) originally described from *Salmonella* spp., *Escherichia coli/Shigella, Helicobacter* spp., *Streptococcus pneumoniae*, and *Staphylococcus aureus* (Table 1).

**Table 1:** **Bacterial human virulence genes used for TBLASTN analysis of environmental metagenomic datasets**.

**Virulence factor**	**Gene**	**Protein size (aa)**	**Reference**
Shiga-like toxin 1	*stx1*	315	ACU32680.1
Shiga-like toxin 2	*stx2*	319	YP_003234845.1
Vacuolating cytotoxin	*vacA*	1296	YP_002301516.1
Haemolysin A	*hlyA*	1024	NP_755445.1
Hyaluronidase	*hylA*	1066	NP_344851.1
Pneumolysin	*ply*	471	NP_346351.1
Gamma-haemolysin component C precursor	*hlgB*	325	YP_041861.1
P fimbriae	*papC*	839	NP_755465.1
P fimbriae	*papA*	199	NP_755467.1
P fimbriae	*papH*	195	NP_755466.1
Type 1 fimbriae	*fimH*	303	NP_757248.1
F1C fimbriae	*focG*	167	NP_753158.1
Type III, secretion apparatus	*invA*	685	YP_151923.1
Type III, secretion apparatus (SPI^a^-2)	*spiA*	497	YP_150710.1
Type III, secretion apparatus (SPI^a^-2)	*ssaD*	403	YP_150709.1
Type III, translocators and effectors (SPI^a^-1)	*sipB*	593	YP_151912.1
Type III, translocators and effectors (SPI^a^-1)	*sipC*	409	YP_151911.1
Regulator of virulence determinants	*phoP*	224	YP_150858.1
Regulator of virulence determinants	*phoQ*	487	YP_150859.1
Part of O-antigen 111	*wbdI*	149	AAC44881.1
Part of O-antigen 157	*rbfE*	364	NP_310868.1
L-Ara4N transferase	*pqaB* (*arnT* region)	548	YP_149876.1
L-Ara4N transferase	*yfbI* (*arnT* region)	550	NP_754685.1
Penicillin-binding protein	*mecA*	668	YP_039515.1

a *Salmonella Pathogenicity Island (SPI)*.

The metagenomes represented terrestrial, marine, limnic as well as several extreme environments (Supplementary Table 1). Environments potentially impacted by human or livestock discharges were excluded from the analysis as were metagenomes restricted to specific bacterial groups.

Default NCBI blastall parameters (expected threshold: 10; word size: 3; matrix: BLOSUM62; gap open cost: 11; gap extend cost: 1) were applied. Hits with an expect (*E*) value < 10^−6^ and a sequence similarity >30% were considered to be significant (Persson et al., 2009). We further applied the criterium of a minimal length of 35 amino acids (aa) to weed-out the shortest 454 pyrosequencing sequences. Longest TBLASTN hits were aligned against the query sequences, and query coverage and percent aa identity determined. As a final criterium to confirm that the environmental hits indeed matched known classes of virulence genes, NCBI BLASTP searches (default parameters) were done using environmental hits as queries.

### Blast searches for clinically relevant bacteria in metagenomic datasets

Bacterial groups from which the virulence genes were originally described (*Salmonella* spp., *E. coli/Shigella, Helicobacter* spp., *Streptococcus pneumoniae* and *Staphylococcus aureus*) were targeted by BLASTN (default parameters, *E*-value < 10^−6^) in the metagenomes. Partial 16S/23S rRNA gene sequences usually amplified with published PCR primers were used as queries (Supplementary Table 2). BLASTN hits were checked for presence of the primer sequences as well as for similarity to the 16S or 23S gene sequences of the clinical bacteria.

### Phylogenetic analysis of virulence gene homologs

Phylogenetic trees showing the relationships between the translated environmental sequences and the corresponding sequences of the clinical genes, were prepared with MEGA Version 5.01 (Tamura et al., 2011) using the Minimum Evolution algorithm (pairwise deletion). In addition, the Maximum Parsimony method of MEGA Version 5.01 was employed to create phylogenetic trees (complete deletion; 500 replicates) to infer the evolutionary history of the homologs in comparison with the clinically relevant genes.

### Statistical analysis

To look for trends that potentially could explain differences between metagenomes with respect to presence of virulence gene homologs, a Principal Component Analysis (PCA) was carried out. The PCA was performed with the Matlab based chemometric software package Latentix 2.0 (Latent5 Aps, Copenhagen N, Denmark, www.latentix.com) using the data set shown in Table 2. A PCA model, *X*, was built as the product of an I × K column-wise orthogonal score matrix T and the transpose of the column-wise orthogonal J × K loading matrix P, where I denotes the number of samples (metagenomes), J the number of variables (virulence gene homologs), and K the number of principal components. E is the matrix of model residuals of size I × J (Equation 1). Zeros were inserted in *X* if genes were not detected in a specific environment.

(1) $$X=TP^{T}+E$$

**Table 2:** **Frequency of putative bacterial human virulence genes in environmental metagenomic datasets**.

**Metagenome**	**Toxin**		**Adhesin**					**Secretion**		**Regulatory**		**Inflammatory**		**Resistance**			**Genes detected**
	***hlyA***	***hylA***	***papA***	***papH***	***papC***	***focG***	***fimH***	***invA***	***spiA***	***phoP***	***phoQ***	***rbfE***	***wbdI***	***pqaB***	***yfbI***	***mecA***	
Microbialites (Marine microbial mat)								20									1
FLAS (Saltern)											420	840					2
LineIsland (Seawater)										4^*^		4^*^					2
PeruMarginSediment (Seawater)								16				16					2
Mountain lake (Freshwater)								37		74		258					3
GeneExpression (Seawater)								81^*^		162^*^		162^*^					3
Contaminated soil	23									69		46					3
AmazonRiverPlume (Estuarine)								58		43	1	41^*^					4
Sapelo2008 (Seawater)								186		127	17	214					4
SargassoSea (Seawater)								498		134		197^*^	8^*^				4
DeepMed (Seawater)	555									416		1527				139	4
Yellowstone (Hot spring)										3554	135	779				34	4
AcidMine (Biofilm)								212		734	28	295				34	5
PacificOcean (Seawater)	4^*^							106		85	1	51^*^					5
PBSM (Marine beach sand)	167							167		834		500			167		5
MontereyBay transect (Seawater)								33		101	2	512	2			6	6
HOT:454 (Seawater)								37		177	4	956	5			10	6
N. Pac. Line67 (Seawater)	1							15		40		29	2		1		6
BotanyBay:Sanger (Seawater)								13	3	764	81	527^*^				61	6
AlvinellaPompejana (Hydrothermal vent)								10	4	286		1078			28	65	6
HydrothermalVent	101							182		706	40	948				20	6
TermiteGut					16			246		410	66	689				98	6
SalternMetagenome					5^*^			7^*^		35^*^	14^*^		2^*^	5^*^	14^*^		7
MontereyBay (Seawater)								123		337	7	88	4		4	21	7
GuaymasBasin (Hydrothermal Vent)	1							49		122	27	729	8			34	7
Bacterioplankton (Seawater)	3							110	3	250	3	503	3			7	8
BATS (Seawater)	1				1			51		151	3	445	2			6	8
BermudaOceanic (Seawater)	4							92		454	33	1141		2	4	122	8
IceMetagenome (Freshwater ice)					4		4	138		960	13	605		13	21		8
HypersalineMat (Seawater)	83^*^							142^*^		1460^*^	107^*^	973^*^	12^*^		12^*^	71	8
CellCapture (Marine sediment)					63			333	10	1553	135	1053		156	177	42	9
Drifting-ESP (Seawater)	0.45				0.45		0.23	21		58	1	152	2			2	9
GutlessWorm	16		3					92		658	48	705		10	32	86	9
HOT:Sanger (Seawater)	8							61	1	653	26	1000	11	1	11		9
WesternChannelOMM (Seawater)					5^*^			71	1	332	27	787^*^	14^*^	4	4^*^	56	10
MILOCO:454Shotgun (Seawater)	0.25^*^	0.06						85	1	275	16	842^*^	10^*^	0.19	1^*^	71	11
Washington Lake (Freshwater)	88	5						126	14	1643	144	824	19	23	42	84	11
BotanyBay:454 (Seawater)	2^*^				1^*^			33	1	414	19	352^*^	1^*^	1	1^*^	54	11
AntarcticaAquatic:Shotgun (Seawater)	1			1				41	2	36	7	661	3	1	3	24	11
YLAKE (Hot spring)	0.25	6			1			69	0.12	427	7	543	1	4	8	47	13
AntarcticaAquatic:454 (Seawater)	0.44	0.04			0.13			60	1	250	15	783	4	4	4	52	13
BisonMetagenome (Hot spring)	11^*^		2^*^		95^*^	8^*^		144^*^	11^*^	1242^*^	47^*^	679^*^	6^*^	28^*^	83^*^	44	13
MILOCO:454 (Seawater)	0.08^*^		1^*^		18^*^	2^*^	4^*^	19	2	80	7	136^*^	1^*^	8	10^*^	5	14
GOS (Seawater)	4^*^	0.08	4^*^		19^*^	0.16^*^	0.16^*^	92	5	685	53	902^*^	4^*^	3	6^*^	5	16
DayNight (Seawater)																	0
SapeloIsland (Seawater)																	0
No. of metagenomes	24	5	4	1	13	3	4	40	16	42	34	43	22	17	22	29	

*The prevalence of the individual genes in the different metagenomes were normalized to the metagenomic library sizes, i.e., reported values are frequencies per billion bases sequenced. Virulence genes not detected in any of the metagenomic datasets were ply, vacA, stx1, stx2, hlgB, sipB, sipC, and ssaD. Please refer to Supplementary Table 1 for a description of the metagenomes*.

* *Detection (BLASTN; E-value < 10^−6^) of partial 16S/23S rRNA genes of clinical strains known to carry the virulence genes*.

The data set was autoscaled across metagenomes (i.e., the mean value of a variable across all metagenomes was subtracted from the data set (mean-centered) and the mean-centered data divided by the standard deviation) prior to PCA. Autoscaling was needed to allow each virulence gene to contribute equally to the PCA model, independent of the overall abundance of each gene. The model was validated by cross-validation (Martens and Martens, 2001).

Only metagenomes consisting of subsamples from similar environments were included in the analysis, hence, 10 metagenomes were excluded (MontereyBay transect, BotanyBay:Sanger, GuaymasBasin, MILOCO:454Shotgun, HOT:Sanger, AntarcticaAquatic:454, BotanyBay:454, AntarcticaAquatic:Sanger, MILOCO:454, GOS). Furthermore, one of the observed 16 virulence genes (*papH*) was not included the PCA as it was not found in any of the 34 metagenomes included in the analysis.

## Results and discussion

Homologs of 16 of the 24 investigated bacterial human virulence genes were identified in the metagenomic datasets (Table 2). The genes represented toxin, adhesin, secretion system, regulatory, inflammatory, and resistance virulence groups involved in urinary tract infections, gastrointestinal diseases, skin and foreign body infections, and systemic infections. To confirm that environmental homologs matched known virulence factors in the databases, we took the longest environmental homologs with the highest identity to the virulence genes and did a NCBI BLASTP search using the environmental sequences as queries. The environmental sequences matched relevant virulence groups in 95% of the cases (Supplementary Table 3).

**Figure 1:**
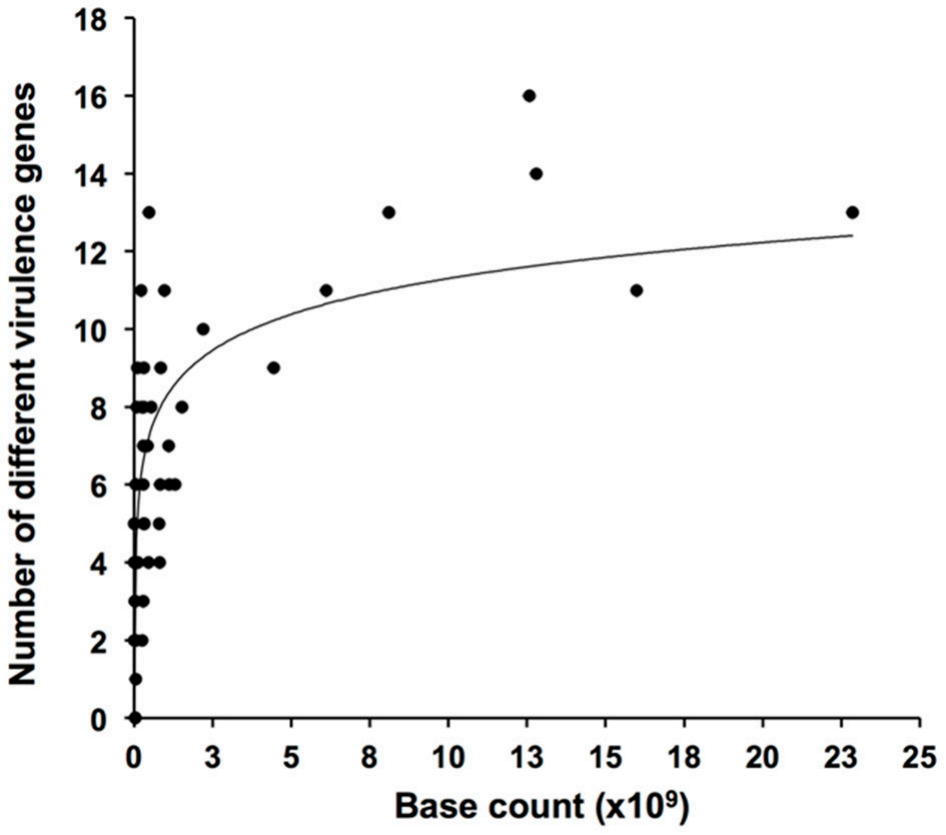
**Rarefaction plot showing numbers of detected virulence genus as function of size (numbers of bases sequenced) of metagenomic datasets**.

A positive relationship between numbers of detected genes and metagenome size was observed (Figure 1). Highest numbers of observed genes were seen in some of the largest metagenomes, but sequencing of more than 3–4 Gb generally did not result in the detection of further genes. Several of the investigated metagenomes were obtained by 454 pyrosequencing with average sequence reads of 100–400 bp. Some metagenomes were based on Sanger sequencing or on assembled sequences with average reads of 600–1000 bp (22). Since short reads (≤ 400 bp) tend to miss a significant amount of the BLAST homologs detected by long reads (Wommack et al., 2008), this may explain the high number of homologs detected in some of the relatively small Sanger-based metagenomes such as the “HydrothermalVent” (50 Mb) (Table 2 and Supplementary Table 1).

The most frequently observed virulence gene homologs were the inflammatory O-antigen gene, *rbfE*, the regulator gene, *phoP*, and the Type III secretion apparatus gene, *invA*, which were detected in ≥85% of the metagenomes (Table 2). These genes also showed the highest relative abundance within the individual metagenomes (Table 2). Homologs to the regulator gene *phoQ*, the toxin gene *hlyA*, the fimbrial gene *papC*, the Type III secretion apparatus gene *spiA*, the inflammatory O-antigen gene *wbdI*, and the resistance genes *pqaB, yfbI* and *mecA* were also found widely distributed (28–72% of the metagenomes). On the other hand, homologs to the fimbrial genes *papA, papH, fimH*, and *focG* and the toxin gene *hylA* were only found in 2–10% of the metagenomes (Table 2).

To assess if the origin of the virulence gene homologs was truly environmental or due to the presence of the clinically relevant bacteria from which the virulence factors were originally described, partial 16S or 23S rRNA gene sequences of *Salmonella* spp., *E. coli, Helicobacter* spp., *Streptococcus pneumoniae* and *Staphylococcus aureus* (Supplementary Table 2) were targeted by BLASTN in the metagenomes. Although we specifically avoided metagenomes influenced by livestock and human discharges, relevant bacterial groups and their respective virulence genes co-occurred 66 out of the 313 times a virulence gene homolog was observed (Table 2). Only *Salmonella* spp., *E. coli*, and *Helicobacter* spp. were observed. The presence of the bacterial groups may be explained by contamination during sample handling, survival and spreading from farms and wastewater treatment plants, or by the natural occurrence of environmental strains of the bacteria. We argue that the presence of the bacterial groups most likely is due to their natural occurrence. For instance, environmental survival of *E. coli* and *Salmonella* is well documented (Maugeri et al., 2004; Miller et al., 2006) and may be occurring in e.g., coastal environments. However, *E. coli* and *Salmonella* were also identified in oceanic, hot spring, and hypersaline environments (Table 2), and albeit contamination by these bacteria cannot be ruled out, it does suggest that they naturally inhabit “unexpected” environments. If, on the other hand, it is assumed that the observation of clinically relevant bacteria is caused either by contamination or by livestock and human discharges, and all cases of dual presence of virulence genes and bacteria are excluded from the analysis, 80% of the detections of virulence gene homologs would still be unbiased by the presence of clinically relevant bacteria. Since 16S/23S rRNA genes contrary to virulence genes often are found in multiple copies per cell (Vos et al., 2012), the risk that 16S/23S rRNA genes of clinically relevant bacteria were overlooked in the metagenomic datasets is miniscule. Thus, the large majority of our data supports the existence of a natural environmental reservoir of genes normally associated with human pathogenic bacteria.

### Distribution of virulence gene homologs across environmental metagenomes

**Figure 2:**
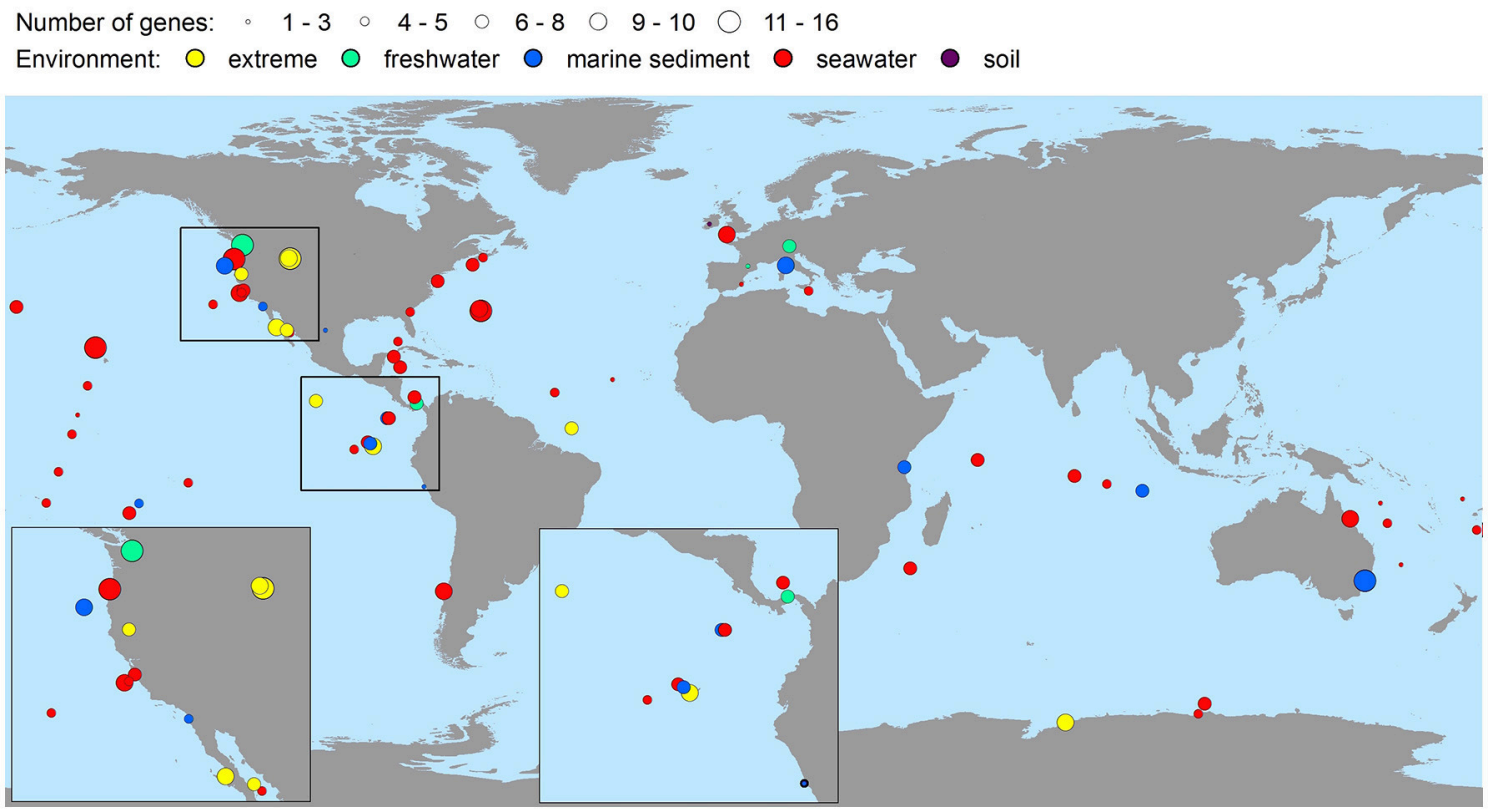
**Global richness map of putative bacterial human virulence genes in environmental metagenomic datasets**. Circles represent findings of genes, and areas of circles the number of virulence genes detected.

The bacterial virulence gene homologs were present in virtually all the metagenomes representing soil, seawater, freshwater, marine sediment, hot spring, the deep-sea, hypersaline mats, microbialites, gutless worms, and glacial ice environments (Table 2). Hence, environments were characterized by highly contrasting environmental conditions in terms of pH, pressure, temperature, salinity, and nutrient availability. Moreover, the virulence gene homologs were globally distributed on six continents and in four oceans (Figure 2), i.e., all continents and oceans for which relevant metagenomes were available. The maximal number of different genes detected within one metagenome was 16 (Table 2). Only in two metagenomes were no genes detected (Table 2). These metagenomes originated from surface water off Hawaii, and coastal sea water off the Sapelo Island (USA) (Table 2 and Supplementary Table 1). The fact that more than half of the sequences of the Hawaiian metagenome were cyanobacterial may explain the lack of virulence gene homologs in this metagenome. With respect to the Sapelo Island metagenome, homologs to virulence genes were observed in another and larger metagenome from Sapelo Island (the Sapelo2008 metagenome; Table 2). Thus, the lack of virulence gene homologs in the SapeloIsland metagenome was most likely due to its small size.

**Figure 3:**
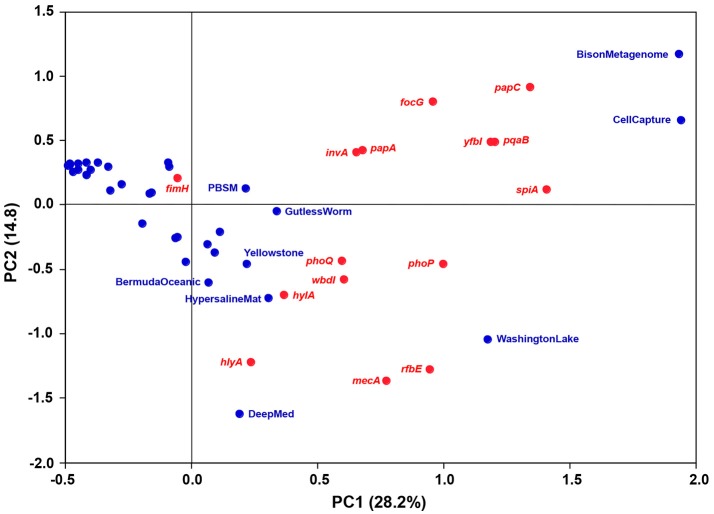
**Bi-plot of principal component 1 (PC1; 26.4% explained variance) and 2 (PC2; 14.2% explained variance) of the PCA model**. Metagenomes are marked in blue, while virulence gene homologs are marked in red.

Metagenomes representing comparable environments (e.g., seawater) did not share a set of typical or common virulence gene homologs. To discern relative differences between the metagenomes with respect to occurrence and frequency of the gene homologs, a principal component analysis (PCA) was performed. The variation explained by the first two principal components was relatively low (43%) yet useful for describing correlations between metagenomes and virulence gene homologs (Figure 3). No general trends were observed as the bulk of the metagenomes clustered relatively closely together with low, and in some cases, negative scores for both PC1 and PC2. However, the CellCapture and Bison metagenomes had relatively high positive PC1 and PC2 scores, while a high positive PC1 and a large negative PC2 score were observed for Washington Lake. The DeepMed metagenome, on the other hand, had a low PC1 score and a very large negative PC2 score. The high positive PC1 scores of the adhesion genes *papC* and focG, the resistance genes *pqaB* and *yfbI*, and the secretion gene *spiA*, indicated that these genes were relatively over-represented in BisonMetagenome and CellCapture as compared to the other metagenomes (Figure 3 and Table 2). The Washington Lake metagenome had above average gene frequencies of the regulatory gene *phoP*, the resistance gene *mecA* and the inflammatory gene *rbfE*, whereas the DeepMed metagenome had a relative over-representation of the toxin gene *hlyA* in comparison to the other metagenomes (Figure 3 and Table 2).

Homologs to eight of the investigated virulence genes (*vacA, stx1, stx2, sipB, sipC, hlgB, ply, ssaD)* were not identified in any of the metagenomes. According to the databases, these genes have narrow phylogenetic host ranges, limited to only one or two bacterial classes/families (Søborg et al., 2014; unpublished data). This narrow phylogenetic host range, however, is unlikely to be the reason why homologs were not identified in the metagenomes since e.g., *phoQ* and *yfbI* also have rather limited host ranges (*Gammaproteobacteria*), yet were frequently observed (Table 2). By PCR-based techniques we have previously observed *vacA, stx1*, and *stx2* in various soils and a freshwater biofilm (Søborg et al., 2013) demonstrating that these genes are present amongst non-human environmental microbiomes.

### Potential ecological functions of virulence gene homologs

The ecological function of the virulence gene homologs in environmental bacteria, and why certain homologs seem to be overrepresented in some microbiomes, is uncertain. The ubiquitous presence of the genes suggests they carry important functions, and it has been proposed that they are house-keeping genes essential for survival and proliferation in outer environments (Søborg et al., 2013) for instance by enabling the bacteria to cope with their physico-chemical environment, avoid predation and/or interact with host organisms. Bacterial DNA isolation procedures usually involve a size fractionation step of the environmental sample prior to DNA extraction. Thus, with the exception of the termite gut, the gutless worm and the Alvinella pompejana metagenomes it is not likely that DNA of bacteria associated with large multicellular organisms is represented in the metagenomes. This could suggest that the primary function of the virulence gene homologs is unrelated to interactions with multicellular host organisms. However, since free-living and host-associated subpopulations may coexist, one cannot rule out the possibility that the identified virulence gene homologs also play a role in host-pathogen interactions in the outer environment.

To be important for the survival of the bacteria in the environment the virulence gene homologs must be both functional and expressed in the non-host environments. Full-length or almost full-length (>95%) sequences of seven genes (*papA, papC, focG, invA, phoP, rfbE*, and *wbdI*) were observed by single TBLASTN hits. In the case of the *wbdI* gene in the Washington Lake metagenome, 98% of the sequence, including the GDP-mannose binding site, was covered by a single TBLASTN hit (Supplementary Figure 1A). In some cases (e.g., *phoP* and *wbdI*) complete or almost complete sequences were found in several metagenomes. For the genes *papH, spiA, phoQ, pqaB, yfbI* and *mecA*, 65–80% of the complete gene sequence were covered by single TBLASTN hits in at least one metagenome (exemplified by *pqaB*; Supplementary Figure 1B). The fact that full-length genes were frequently observed suggests they are potentially functional and do not just occur as fragmented virulence genes within the microbiomes. As further support we have previously demonstrated *in vitro* expression of *fimH* in natural soil bacterial isolates and the expression of a virulent phenotype in soil bacterial isolates carrying homologs to the hemolysin gene, *hblA*, (Søborg et al., 2014). Some of the metagenomes (DayNight, Contaminated soil, MILOCO454, PacificOcean, and WesternChannel) were metatranscriptomes. Hence, the 15 different virulence gene homologs detected in these metatranscriptomes provide evidence of environmental transcription.

The BisonMetagenome represented samples collected along a thermal gradient (93–56⋅C) of a hot spring (Bison Pool) in Yellowstone National Park (Supplementary Table 1). Theoretically, the bacterial community in Bison Pool may have been contaminated by bacteria from livestock as bison frequenting the pool during winter months may potentially have been in contact with livestock outside the borders of the park. However, since samples were taken at water temperature between 93 and 56⋅C, the risk of contamination is miniscule. Hot spring environments are often characterized by dense biofilms, hence, a relative overrepresentation of adhesin genes (*papC* and *focG*) is intuitively understandable, as fimbriae on the outer surface of the bacteria may play an important ecological role in biofilm formation (Lasaro et al., 2009). The resistance genes *pqaB* and *yfbI* also appeared to be relatively overrepresented in BisonMetagenome. They encode L-Ara4N transferases that add L-Ara4N moieties to lipid A of the cell membrane of Gram-negative bacteria. The modification of lipid A interferes with the recognition by the Toll-like receptor TLR-4 on macrophages/neutrophils and promote resistance toward cationic antimicrobial peptides in phagosomes (Baker et al., 1999; Trent et al., 2001; Miller et al., 2005). Thus, an over-representation of these genes may be explained as an adaptation to resist phagocytosis by thermophilic amebae which in some cases have been shown to be abundant in hot water environments (Tyndall et al., 1989; Reeder et al., 2015). In a similar manner, *spiA* may play a role for the bacteria in their interaction with amebae. In *Salmonella, spiA* encodes a Type III secretion system outer membrane protein, SpiA, involved in the transport of virulence proteins into host cells (Cirillo et al., 1998). Therefore, SpiA, in combination with the L-Ara4N transferases encoded by *pqaB* and *yfbI*, may be involved in the infection (internalization) of amebae.

Homologs to *papC, focG, pqaB, yfbI*, and s*piA* were also overrepresented in the CellCapture metagenome based on samples of Pacific deep-sea sediment (Supplementary Table 1). A plausible explanation for an over-representation of adhesion genes in deep-sea sediment is not straightforward but the resistance genes and the Type III secretion gene may help the bacteria avoid predation by protozoa the same way as in hot springs.

The Washington Lake metagenome had above average frequencies of homologs to the regulatory gene *phoP*, the inflammatory gene *rbfE* and the resistance gene *mecA*. The two-component regulatory system phoP/Q, encoded by *phoP* and *phoQ*, governs adaptation to low Mg^2+^ and Ca^2+^ environments and to intramacrophage survival (Groisman, 2001; Tu et al., 2006), while *rbfE* encodes a perosamine synthetase involved in the synthesis of the O antigen of lipopolysaccharide in the outer membrane of Gram-negative bacteria (Iguchi et al., 2011). The relative over-representation of *phoP* and *rfbE* in the WashingtonLake metagenome suggests they provide specific selective advantages for the bacteria in these two environments. On the other hand, since they encode general house-keeping functions, their presence may not be exceptional. *mecA* encodes a peptidoglycan transpeptidase (with low affinity to ß-lactam antibiotics) involved in cell wall synthesis (Wielders et al., 2002). Although *mecA* may be considered a basic house-keeping gene, the finding of environmental homologs is noteworthy. *mecA* is highly conserved among clinical methicillin-resistant strains of *Staphylococcus aureus* (MRSA) (>90% sequence identity between strains) and encodes a peptidoglycan transpeptidase unlike any of the peptidoglycan transpeptidases normally produced by *S. aureus* (< 21% sequence identity) (Lim and Strynadka, 2002). It consists of two regions with homology to a penicillinase gene and a penicillin-binding protein gene, and has been hypothesized to have evolved by homologous recombination and transferred to MRSA by horizontal gene transfer from an unidentified species (Hiramatsu, 1995; Lim and Strynadka, 2002). Our data suggest that this unidentified bacterial species may have been of environmental origin.

The ecological role of the above average frequency of homologs to the toxin gene *hlyA* in the DeepMed seawater metagenome is somewhat perplexing. *hlyA* encodes a hemolysin (HlyA) involved in the lysis of (red blood) cells. However, marine opportunistic fish and shellfish pathogens such as Vibrios may produce hemolysins, which share common features with the HlyA hemolysin (Zhang and Austin, 2005; Ruwandeepika et al., 2010). Hence, the bacteria carrying homologs to *hlyA* may possibly be involved in the pathogenesis of marine animals. Another potential role of hemolysin could be as an antipredator defense strategy to subvert phagocytosis similar to the group B streptococcal β-hemolysin/cytolysin encoded by the *cylE* gene (Liu et al., 2004).

### Phylogenetic relationships between virulence genes and environmental homologs

Similarities >50% between clinical and environmental sequences were observed for eleven genes (*hlyA, papC, fimH, invA, phoP, phoQ, pqaB, rfbE, wbdI, yfbI* and *mecA*) and for 6% of all hits. In four cases (*fimH, phoP, phoQ* and *yfbI*) were an identity of more than 90% observed between the environmental and clinical sequences (Supplementary Table 3). The results concur with a previous study in which sequence similarities of 90–100% between environmental and clinical virulence genes were found (Søborg et al., 2013), and taken together emphasize that sequence conservation between environmental and clinical genes may be high.

**Figure 4:**
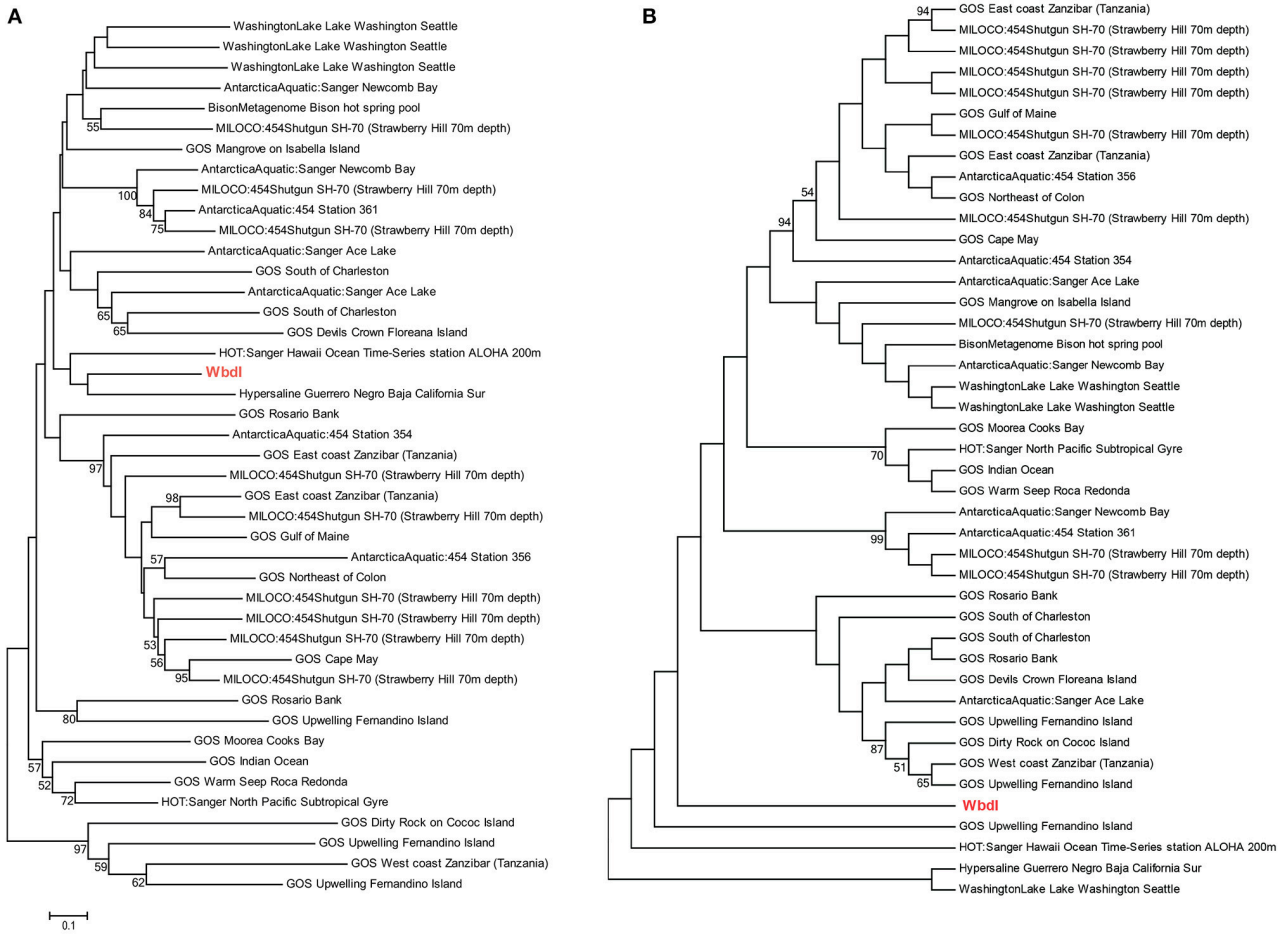
**Minimum evolution distance tree (A) and maximum parsimony tree (B) of environmental sequences homologous to the protein sequence of *wbdI.*** Only environmental sequences with a coverage >95% (>141 aa) are included. The clinical sequence of wbdI is shown in red boldface type. Bootstrap values above 50% are displayed.

**Figure 5:**
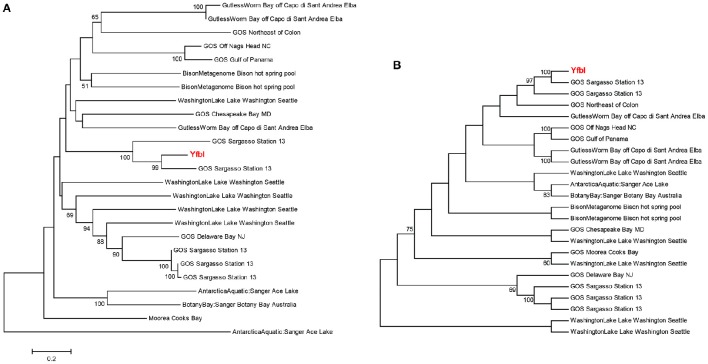
**Minimum evolution distance tree (A) and maximum parsimony tree (B) of environmental sequences homologous to the protein sequence of *yfbI***. Only environmental sequences with a coverage >40% (>220 aa) are included. The clinical sequence of yfbI is shown in red boldface type. Bootstrap values above 50% are displayed.

Phylogenetic analyses underlined the close relationship between clinical genes and environmental homologs, and demonstrated relatively large sequence variations between environmental homologs with no clear relationship between environment and phylogeny (Figures 4A, 5A and Supplementary Figure 2). For instance, the phylogenetic distance between the clinical sequence of WbdI and its most closely related environmental homolog (HypersalineMat metagenome), and the distance between YfbI and its most closely related environmental neigbor (GOS Sargasso Station 13 metagenome), were both 0.7 (Figures 4A, 5A). In contrast, phylogenetic distances up to 1.8 and 2.2 were observed between the environmental homologs to WbdI and YfbI, respectively. Furthermore, distances between the environmental sequences were larger than the largest distances between the clinical sequences and their environmental homologs (Figures 4A, 5A and Supplementary Figure 2).

Examination of the evolutionary history of the virulence genes indicated that the environmental homologs in some cases represented ancient lineages in the phylogeny of the clinical genes (Figures 4B, 5B and Supplementary Figure 2). In the case of YfbI, all environmental sequences were evolutionary basal to the clinical sequence (Figure 5B), while several of the environmental homologs to WbdI, PapA and HlyA showed deep branching positions relative to the clinical sequences (Figure 4B and Supplementary Figure 2). This suggests that the environmental genes in some cases evolved relatively early in the phylogeny of the clinical genes and that the origin of virulence genes in clinically relevant bacteria may be environmental.

## Conclusion

Our findings highlight the widespread occurrence and transcription of genes often associated with bacterial human pathogens across diverse natural (non-human) environments. Environmental bacteria carrying homologs to virulence genes are probably not opportunistic human pathogens. Most likely, the homologs to the virulence factors are maintained because of the advantages they provide for the survival of the bacteria outside the host environment (Brown et al., 2012). Pathogenesis may, therefore, evolve by “coincidental” selection in non-host environments because traits that confer virulence in a host environment may also be beneficial for bacteria in non-host environments (Mikonranta et al., 2012). The high degree of sequence conservation between some of the environmental and clinical genes, and the phylogenetically deep branching positions of the environmental sequences, suggest that the environmental genes are precursors of virulence genes found in clinically relevant bacteria. Hence, our results suggests that coincidal selection in outer (non-host) environments may be a source of virulence traits for opportunistic pathogenic bacteria.

## Author contributions

DS, NH, MK, and NK conceived and designed the research project and contributed with the interpretation of results. JC performed the statistical analysis. DS and NK wrote the manuscript which was approved by all authors.

### Conflict of interest statement

The authors declare that the research was conducted in the absence of any commercial or financial relationships that could be construed as a potential conflict of interest.
