# Supplementary material for: Bacterial Human Virulence Genes across Diverse Habitats As Assessed by In silico Analysis of Environmental Metagenomes
Source: Front Microbiol. 2016 Nov 3;7:1712. doi: 10.3389/fmicb.2016.01712 (PMC5093120; doi:10.3389/fmicb.2016.01712)
Supplement: Supplementary file 1 [file Table1.PDF]

Supplementary Table 1. **Metadata describing the metagenomic datasets used for assessing the environmental occurrence of bacterial human virulence genes (http://camera.calit2.net/camdata.shtm; http://data.imicrobe.us).**

Metagenome	Description	Base count	Sequence count	Range of average read lengths
AcidMine: All Metagenomic Sequence Reads (N) (NCBI Bioproject PRJNA208477)	Acid mine drainage biofilm metagenome, Richmond Mine, Iron Mountains (elevation ~ 950m), California, from the 5-way (acid mine drainage superfund site, pH 0.83, 42°C) and Ultra Back A (pH 1.28, 38°C) sites. Biofilms were dominated by a small number of species and contained mixtures of bacteria (*Leptospirillum*, *Sulfobacillus* and, in a few cases, *Acidimicrobium*) and archaea (*Ferroplasma* and other members of the *Thermoplasmatales*).	325,778,024	319,166	1004.75 - 1041.56
AcidMine: Assembled Sequences (N)	Scaffolds assembled from Acid Mine samples	23,533,043	3,466	-
AlvinellaPompejana: All Metagenomic Sequence Reads (N) or EpibiontMetagenome: All Metagenomic Shotgun Reads (N) (NCBI Bioproject PRJNA17241)	*Alvinella pompejana* epibionts collected from a hydrothermal vent in the East Pacific Rise at a depth of 2500m. They are a multispecies consortium of *Epsilonproteobacteria* present as a biofilm (>95% *Proteobacteria*). They harbor wide-ranging adaptive traits that include high levels of strain variability analogous to *Epsilonproteobacteria* pathogens such as *Helicobacter pylori*, metabolic diversity of free-living bacteria, and numerous orthologs of proteins that are hypothesized to be optimally adapted to specific temperature ranges in the habitat. The habitat is characterized by a steep thermal gradient (20-80°C), little to no oxygen, and high concentrations of heavy metals which likely impose strong selective pressures on the symbiont/host association.	290,371,756	293,065	990.81
AmazonRiverPlume: All Metagenomic 454 Reads (N) (iMicrobe project: CAM_PROJ_AmazonRiverPlume)	Microbial community gene expression across a productivity gradient of the Amazon River plume (across the Amazon River plume to the eastern Equatorial Atlantic Ocean). The equatorial Atlantic Ocean is greatly influenced by freshwater inputs from the Amazon and Orinoco River plumes in the west, upwelling on the western boundary of Africa, and Aeolian dust inputs throughout the basin. The region is characterized by a strong east-west gradient in water temperature and productivity, where warm lower-salinity waters in the western basin merge with cooler nutrient poor waters of the eastern basin within the equatorial counter-current.	812,890,804	3,856,789	135.39 - 287.87
AntarcticaAquatic: All Metagenomic 454 Reads (N) (NCBI Bioproject PRJNA33179)	Samples were collected in December 2006 from two marine-derived meromictic lakes (Ace Lake and Organic Lake) in the Vestfold Hills region of East Antarctica and ocean samples in	22,853,230,277	63,659,807	277.78 - 409.46

Metagenome	Description	Base count	Sequence count	Range of average read lengths
	the vicinity of Casey Research Station. DNA was isolated from the 0.1 to 0.8 microns size fraction. Ace Lake is the most intensively studied stratified lake in Antarctica. The saline stratification maintains zones that are aerobic, through to methane-saturated anoxic bottom waters. Organic Lake is also a stratified, cold environment that supports growth of aerobic and anaerobic microorganisms, but unlike Ace Lake it is highly saline.			
AntarcticaAquatic: All Metagenomic Shotgun Reads (N) (NCBI Bioproject PRJNA33179)	-	966,394,509	966,458	971.45 - 1012.24
Bacterial gene expression of a mountain lake: All Metagenomic 454 Reads (N) (NCBI Bioproject PRJEA46645)	Diel gene expression profiles (day and night) of the bacterial planktonic assemblage of a phosphorus limited mountain lake Llebreta (1620 m above sea level) in the Limnological Observatory of the Central Pyrenees, Spain. Dominated by the three most common freshwater phyla: *Bacteroidetes* and *Betaproteobacteria*, each accounting for > 30% of total amplicons, and *Actinobacteria*, accounting for > 20%. *Alphaproteobacteria* and *Gammaproteobacteria* were notably less abundant.	27,101,336	151,484	167.41 - 191.08
Bacterioplankton: All Metagenomic 454 Reads (N) (iMicrobe project: CAM_PROJ_Bacterioplankton)	Surface water microbial community samples from the 5 - 0.2 micron size fraction taken at 7 locations between Fiji and Hawaii. The samples range from the South Pacific Subtropical Gyre, across the region of equatorial upwelling, into the North Pacific subtropical gyre. In addition, an equatorial sample in the > 5 micron size fraction was sequenced. The equatorial Pacific Ocean is an ideal location for examining the influence of variable organic matter quantity upon activities of tropical surface water microorganisms.	300,253,302	1,314,590	205.11 - 256.7
BATS: All Metagenomic 454 Reads (N) (NCBI Bioproject PRJNA40125)	BATS (Bermuda Atlantic Time-series Study), is located in the northwestern region of the Sargasso Sea, an oligotrophic ocean gyre. Gyres, which cover 70% of the ocean surface, are low in surface nutrients and productivity. At BATS, cool winter weather and large storms cause deep winter mixing of the water column, bringing nutrients to the surface that stimulate spring phytoplankton blooms, which are followed by summer lows in productivity.	1,517,015,244	6,784,781	182.7 - 243.34
Bermuda Oceanic Microbial Observatory Course: All Metagenomic	Metagenomes for the 2010 Bermuda Institute of Ocean Sciences Microbial Oceanography Course.	541,669,011	1,297,714	413.96 - 421.03

Metagenome	Description	Base count	Sequence count	Range of average read lengths
454 Reads (N) (iMicrobe project: CAM_P_0000712)				
BisonMetagenome: All Metagenomic Shotgun Reads (N) (NCBI Bioproject PRJNA36675)	Samples were collected along a thermal gradient at a hot spring in Yellowstone National Park called Bison Pool. The individual samples range from the boiling source pool to a downstream photosynthetic mat.	472,709,986	476,083	888.61 - 1144.93
BotanyBay: All Metagenomic 454 Reads (N) (NCBI Bioproject PRJNA34751)	Metagenome of Botany Bay, Australia.	6,117,299,693	15,232,931	248.96 - 509.02
BotanyBay: All Metagenomic Sanger Reads (N) (NCBI Bioproject PRJNA34751)	-	307,528,657	305,600	971.18 - 1067.19
CellCapture: All Metagenomic 454 Reads (N) (iMicrobe project: CAM_PROJ_CellCapture)	Syntrophic anaerobic methane oxidizing ANME-2c archaea and physically associated microorganisms directly from deep-sea marine sediment were collected directly from the environment (Eel River Basin, United States) using combined fluorescence in situ hybridization and immunomagnetic cell capture. These consortia are globally distributed in the environment and provide a significant sink for methane by substantially reducing the export of this potent greenhouse gas into the atmosphere. An unexpected diversity of associated bacteria, including *Betaproteobacteria* and a second sulfate-reducing *Deltaproteobacterial* partner was revealed.	95,952,116	501,678	168.93 - 218.43
DayNight: All Metagenomic 454 Reads (N) (NCBI Bioproject PRJNA33463)	Microbial assemblages (< 5 mm) from surface water at the Hawaiian Ocean Time-Series (HOT) revealed community-wide metabolic activities and day/night patterns of differential gene expression. *Cyanobacteria* contributed a greater percentage of the transcripts (54% of annotated sequences). Major heterotrophic taxa contributing to the community transcriptome included *Alphaproteobacteria* (19% of annotated sequences) and *Gammaproteobacteria* (4%).	28,618,075	289,539	98.2 - 99.28
DeepMed: All Metagenomic Sequence Reads (N) (NCBI Bioproject PRJNA19587)	Study of the bathypelagic region at Ionian KM3 station located over the Ionian abyssal plain, a flat deep basin occupying most of the space between Sicily and Greece in the Eastern Mediterranean. The deep waters of the Mediterranean are special in being free from the intrusion of polar waters that feed most the bottom of the global ocean. The Ionian sample comes from 3000 m deep and is submitted to a continuous	7,203,198	9,048	796.11

Metagenome	Description	Base count	Sequence count	Range of average read lengths
	pressure of 300 Kg/cm^2 but contrastingly to most deep ocean habitats this has a relatively warm temperature of nearly 14°C.			
Drifting_ESP_Coastal_Pacific_September_2010: All Metagenomic 454 Reads (N) (iMicrobe project: CAM_P_0001026)	Lagrangian sampling of Monterey Bay, California surface waters. The Environmental Sample Processor, a robotic environmental sampler and sensor, was used to collect and preserve microbial cells from a depth of approximately 23 m. The instrument was deployed on a drifting platform to facilitate Lagrangian sampling and enable continuous tracking and repeated sampling of coherent microbial populations. Over the course of a 5-day deployment, the instrument drifted approximately 80 km along the warm side of a front that was generated by coastal upwelling to the east. Thirteen samples collected at four-hour intervals were selected for sequencing.	4,436,185,819	9,985,281	433.46 - 453.92
FLAS: All Metagenomic Sequence Reads (N) (Genbank: DU824018 - DU826964)	Metagenome from a marine solar saltern in Alicante, Spain. It is a hyper-saline environment generated by evaporation of seawater isolated under arid conditions. As sea water concentrates, biological diversity starts a slow decrease that culminates at values around 10 times typical marine levels (about 400g of salts per liter of water).	2,380,900	2,947	807.91
GeneExpression: All Metagenomic 454 Reads (N) (iMicrobe project: CAM_PROJ_GeneExpression)	Surface water microorganisms were collected in the equatorial North Atlantic ocean and South Pacific Subtropical gyre, United States. Because sunlight exerts profound influence upon biogeochemical processes in the upper ocean (e.g. photosynthesis and nitrogen fixation), samples were collected in both light and dark phases to compare diel gene expression patterns.	283,285,834	1,525,824	135.9 - 224.61
GOS: All Metagenomic Sequence Reads (N) (NCBI Bioproject PRJNA13694)	A global circumnavigation aboard the Sorcerer II sailing yacht began in August 2003, starting in Halifax, Canada and samples were collected at sites along the U.S. east coast, Gulf of Mexico, Galapagos Islands, central and south Pacific Oceans, Australia, Indian Ocean, South Africa, across the Atlantic back to the U.S., and was completed in January 2006. The samples have produced the largest catalogue of genes to date from thousands of known and new species.	12,578,058,565	13,634,249	352.03 - 402.73 (NGS)* 826.5 - 1142.02 (Sanger)
GutlessWorm: All Metagenomic Sequence Reads (N) (NCBI Bioproject PRJNA17779)	Juvenile and adult specimens of the marine oligochaetes *Olavius algarvenis* and *Olavius ilvae* (gutless worm lacking a mouth, gut and nephridia) were collected from silicate sediments in a bay off Capo di Sant' Andrea, Elba, Italy. Four co-occurring bacterial symbionts were identified and shown to	314,746,819	313,773	1003.1

Metagenome	Description	Base count	Sequence count	Range of average read lengths
	be capable of carbon fixation, which provides the host with multiple sources of nutrition.			
GutlessWorm: Assembled Sequences (N)	Scaffolds assembled from Gutless Worm samples.	60,554,445	6,714	-
Guaymas Basin deep-sea Metagenome (N) (NCBI Bioproject PRJNA77837)	Deep-sea hydrothermal plumes and background seawater of Guaymas Basin and Carmen Basin in the Gulf of California. Guaymas Basin plumes represent biogeochemical hot spots where microorganisms are stimulated by hydrothermal inputs (methane, ammonium, sulfur, manganese) and mediate biogeochemical reactions. The Guaymas Basin was dominated by *Alpha*- and *Gammaproteobacteria* and contained 163 bacterial OTUs.	1,105,246,296	4,970,673	139.58 - 355.36
HOT: All Metagenomic 454 Reads (N) (NCBI Bioproject PRJNA29033)	From different depth profiles at the North Pacific Subtropical Gyre at station ALOHA. Upper depth strata are characterized by steep gradients in light quality and intensity, temperature, and macronutrient and trace metal concentrations. At greater depths, low temperature, increasing hydrostatic pressure, the disappearance of light, and dwindling energy supplies largely influence oceanic biota.	828,623,247	4,760,479	102.19 - 231.78
HOT: All Metagenomic Sequence Reads (N) (NCBI Bioproject PRJNA29033)	-	846,254,817	926,772	876.6 - 1028.97
HydrothermalVent: All Metagenomic Shotgun Reads (N) (NCBI Bioproject PRJNA37895)	Metagenome of thick microbial biofilms consisting of just a few dominant species from the carbonate chimneys of the Lost City Hydrothermal Field on the Mid-Atlantic Ridge. The biofilm had a remarkable abundance and diversity of genes potentially involved in lateral gene transfer. More than 8% of all metagenomic reads showed significant sequence similarity to transposases. Low diversity, a single phylotype belonging to the *Methanosarcinales* order of methane-cycling archaea constitutes >80% of all active cells in the hottest, anoxic zones of the chimney, while a few species of aerobic and microaerophilic bacteria dominate the cooler, oxygenated zones.	49,566,108	49,636	998.59
HypersalineMat: All Metagenomic Shotgun Reads (N) (NCBI Bioproject PRJNA29795)	Metagenomes of 10 successive layers of a phylogenetically complex hypersaline mat from Guerrero Negro, Mexico. Pronounced millimeter-scale genetic gradients were consistent with the physicochemical profile of the mat. Despite	84,253,870	129,147	622.21 - 703.58

Metagenome	Description	Base count	Sequence count	Range of average read lengths
	these gradients, all layers displayed near-identical and acid-shifted isoelectric point profiles due to a molecular convergence of amino acid usage, indicating that hypersalinity enforces an overriding selective pressure on the mat community. *Cyanobacteria* and *Alphaproteobacteria* were the most abundant lineages in the top two layers. Below the upper 2 mm, *Proteobacteria*, *Bacteroidetes*, *Chloroflexi* and *Planctomycetes* were the most represented phyla.			
IceMetagenome: All Metagenomic 454 Reads (N) (NCBI Bioproject PRJNA30701)	DNA was isolated from glacial ice of the Northern Schneeferner, Germany. The *Proteobacteria* (mainly *Betaproteobacteria*), *Bacteroidetes*, and *Actinobacteria* were the predominant phylogenetic groups. In total 108 OTUs were identified.	239,698,813	1,076,539	222.66
LineIsland: All Metagenomic 454 Reads (N) (NCBI Bioproject PRJNA28667)	Microbial communities on four coral atolls in the Northern Line Islands, central Pacific, United States. Kingman, a small uninhabited atoll which lays most northerly in the chain, had microbial and water chemistry characteristic of an open ocean ecosystem. On this atoll the microbial community was equally divided between autotrophs (mostly *Prochlorococcus* spp.) and heterotrophs. In contrast, Kiritimati, a large and populated (~5100 people) atoll, which is most southerly in the chain, had microbial and water chemistry characteristic of a near-shore environment. On Kiritimati, there were 10 times more microbial cells and virus-like particles in the water column and these microbes were dominated by heterotrophs, including a large percentage of potential pathogens (human pathogens like *Staphylococcus*, *Vibrio*, and *Escherichia*, fish pathogens like *Aeromona*, and plant pathogens from the *Xylella* genera). The middle atolls, Palmyra and Tabuaeran, had intermediate densities of microbes and viruses and higher percentages of autotrophic microbes than either Kingman or Kiritimati.	256,355,930	2,428,119	103.37 - 109.95
Metatranscriptomics of contaminated soil: All Metagenomic 454 Reads (N) (iMicrobe project: CAM_P_0000911)	The microbial response to the polycyclic aromatic hydrocarbon Phenanthrene in soil (temperate humid soil, from County Tipperary, Ireland) was evaluated by sequencing of extracted RNA. Annotation showed a marked increase in transcripts involved in aromatic compound metabolism, respiration, stress responses and amino acid metabolism, and concurrent decreases in virulence, motility, chemotaxis, carbohydrate and DNA metabolism transcripts. Taxonomic identity of the most abundant transcript groups revealed that	43,719,012	127,686	342.39

Metagenome	Description	Base count	Sequence count	Range of average read lengths
	the *Actinobacteria* were responsible for most of the de novo expression of dioxygenases, stress response and detoxification genes.			
Microbialites: All Metagenomic 454 Reads (N) (NCBI Bioproject PRJNA28351)	Microbial communities from living microbialites from Cuatro Cienegas, Mexico. The microbialite community is distinct from other marine and freshwater microbial communities, and demonstrates extensive environmental adaptation. Heterotrophic bacteria (69%) dominated the metagenomic sequences from Pozas Azules II, while the Rio Mesquites metagenomes was dominated by *Cyanobacteria* (74%). Among the heterotrophic bacteria at Pozas Azules II, *Alphaproteobacteria*, *Gammaproteobacteria*, and *Planctomycetes* were the most common. The most common cyanobacterial sequences in both microbialite metagenomes were *Nostocales* and *Chroococcales*.	49,697,439	475,529	103.62 - 106.76
MILOCO: All Metagenomic 454 Whole Genome Shotgun Reads (N) (iMicrobe project: CAM_P_0000692)	Microbial Initiative in Low Oxygen areas off Concepción, Chile and Oregon, USA. Naturally occurring regions of hypoxia are found along the Eastern Boundary Current Ecosystems (EBCE). Some of these largest regions, the Oxygen Minimum Zones, are found in the upwelling areas along the North and South Pacific Ocean where their intensity, thickness, and temporal stability varies as a function of latitude. Although EBCE represent less than 5% of the ocean's surface, they support regions of high biological activity that contribute significantly to global elemental cycles.	15,992,730,206	35,614,414	166.88 - 567.83
MILOCO: All Meta-transcriptomic 454 Reads (N) (iMicrobe project: CAM_P_0000692)	-	12,805,967,899	33,645,227	-
MontereyBay: All Metagenomic 454 Reads (N) (NCBI Bioproject PRJNA29037)	Monterey Bay, California is a well-studied coastal environment characterized by strong seasonal upwelling, with active local fisheries and adjacent to one of the most productive agriculture areas in the country, and within the Monterey Bay National Marine Sanctuary. Monthly monitoring cruises transecting the Bay has been conducted over the last 19 years, and microbial fraction samples were synoptically collected over seven of those years (1997-2004). Three surface water samples from mid-bay station M1 were pyrosequenced. These samples were selected based on environmental parameters and on DNA yield, and span October 2000 to May 2001. They	285,012,685	1,200,014	233.45 - 242.16

Metagenome	Description	Base count	Sequence count	Range of average read lengths
	represent non-bloom conditions (10/15/00) and two post-bloom response points (4/25/01 and 5/15/01).			
Monterey Bay transect CN207 Metagenomic 454 Reads (N) (iMicrobe project: CAM_P_0000719)	Microbes from multiple size fractions along a well characterized transect (CN207) from Monterey Bay.	1,110,194,702	5,248,980	171.56 - 244.08
N. Pac. Line67: All Metagenomic 454 Reads (N) (NCBI Bioproject PRJNA16339)	North Pacific metagenomes from Monterey Bay to Open Ocean (CalCOFI Line 67) October 2007. The environmental sequences grouped with pelagophytes, haptophytes, prasinophytes and viruses as well as bacteria. The analyses suggest that multiple independent Pho4 gene transfer events have occurred between marine viruses and both eukaryotic and bacterial hosts.	1,303,972,114	5,618,147	227.27 - 234.27
PacificOcean: All Metagenomic 454 Reads (N) (iMicrobe project: CAM_PROJ_PacificOcean)	Metatranscriptomic samples were collected along a North-South transect in the western South Pacific Ocean, to investigate the relationship between nitrogen-fixing populations and microbial community gene expression in the nitrogen-limited oligotrophic water. Nitrogen-fixer abundance in the region is variable, with blooms and surface aggregations of dominant unicellular and filamentous taxa.	791,429,543	3,953,466	133.19 - 338.65
PBSM: All Metagenomic Shotgun Reads (N) (NCBI Bioproject PRJNA13729)	Silicate-rich, ordinary ocean beach sand was found to concentrate dissolved DNA from seawater over 10,000-fold, providing a rich, renewable, and easily accessible genetic library that is easy to harvest and inexpensive to process. The DNA adsorbed to wet sand from the Pacific Beach in San Diego, California, was sequenced. The modal GC content of the cloned sequences was 61%, with a range of 21%-84%. Open reading frames were found in all sequences, and ranged in size from 21% to 100% of the clone length. 2562 of the 2571 genes found were new, and 2218 encoded proteins that were similar to known proteins. Of these, approximately 90% of the amino acid sequences were similar to gene products from prokaryotic sources, 9% eukaryotic and 1% viral.	5,996,169	4,981	1203.81
PeruMarginSediment: All Metagenomic 454 Reads (N) (Genbank: SRA001015)	Sediments at horizons 1, 16, 32, and 50 m below the seafloor from Ocean Drilling Program Site 1229 on the Peru Margin. The depths include sediments from both primarily sulfate-reducing and methane-generating regions of the sediment column. *Crenarchaeota* are the abundant microbial member.	62,689,920	601,632	103.07 - 105.01
SalternMetagenome: All Metagenomic	Viral (42 viromes) and microbial (45 microbiomes) sequences	431,614,016	4,286,649	361.18 - 417.99

Metagenome	Description	Base count	Sequence count	Range of average read lengths
454 Reads (N)<br>(NCBI Bioproject PRJNA28725)	from salterns of differing salinities in California.			
Sapelo2008: All Metagenomic 454 Reads (N)<br>(NCBI Bioproject PRJNA33823)	Two replicate water samples were collected off the coast of Sapelo Island, in shallow coastal waters of the southeastern United States. The environment is characterized by well mixed and turbid waters that can have dynamic ranges in temperature, salinity, inorganic nutrient concentrations, and dissolved organic matter concentrations. RNA samples were obtained on the night of August 8, 2008 by capturing microbial cells in the 0.2-3.0 micron size range from 10 liters of water.	458,192,991	2,181,899	206 - 213.82
SapeloIsland: All Metagenomic 454 Reads (N)<br>(NCBI Bioproject PRJNA19145)	Bacterioplankton metagenome from coastal sea water samples at the Sapelo Island, United States.	33.375.708	354.908	92.13 - 95.31
SargassoSea: All Metagenomic 454 Reads (N)<br>(NCBI Bioproject PRJNA29043)	Gene expression profiles of a prokaryotic community from surface waters of the western North Atlantic Ocean (BATS station). Samples were enriched with the organic sulfur compound DMSP (25 nM, final concentration) and compared with a no-addition control. Transcripts in the DMSP treatment had more frequent hits to *Gammaproteobacteria*, suggesting that this group might be the first to react when DMSP becomes available. Dominated by rRNA genes from *Proteobacteria* with moderate contributions from *Firmicutes*, *Cyanobacteria*, and species in the CFB phyla (*Cytophaga*, *Flavobacterium*, and *Bacteroides*).	126,606,546	606,285	207.96 - 209.7
TermiteGut: Assembled Sequences (N)<br>(NCBI Bioproject PRJNA337869)	Metagenomic analysis of the bacterial community resident in the hindgut paunch of a wood-feeding *Nasutitermes* species (which do not contain cellulose-fermenting protozoa) from Guapiles, Costa Rica to show the presence of a large, diverse set of bacterial genes for cellulose and xylan hydrolysis. New insights into other important symbiotic functions including H_2 metabolism, CO_2-reductive acetogenesis and N_2 fixation are also provided.	61,000,504	56,249	-
WashingtonLake: Assembled Sequences (N)<br>(NCBI Bioprojects PRJNA30135, PRJNA30133, PRJNA30131, PRJNA30129, PRJNA30127)<br>(iMicrobe project: CAM_PROJ_WashingtonLake)	Metagenome analysis of microbes from Lake Washington in Seattle by labeling DNA through stable isotope probing, followed by whole genome shotgun sequencing.	214,828,794	252,427	-

Metagenome	Description	Base count	Sequence count	Range of average read lengths
WesternChannelOMM: All Metagenomic 454 Reads (N) (iMicrobe project: CAM_PROJ_WesternChannelOMM)	Deep sequence microbial diversity, as well as total community metagenomics and metatranscriptomics, was performed to assess microbial diversity and community function in the Western English Channel. The Western Channel Observatory maintains more than 100 years of continual environmental monitoring, including oceanographic and biological measures. The most abundant OTU belongs to the SAR11 clade and comprised 25% of the community. Bacterial community contained 999 distinct OTUs (based on 16S rRNA sequences).	2,199,020,480	7,354,754	105.13 - 355.63
Yellowstone: All Metagenomic Shotgun Reads (N) (NCBI Bioproject PRJNA20953)	Investigation of a well-studied hot spring microbial mat community in Yellowstone National Park, United States. The mats contain a diversity of microorganisms ranging from phototrophs such as *Cyanobacteria* (predominantly *Synechococcus* spp.) and green non-sulfur-like bacteria such as *Roseiflexus* spp. and *Chloroflexus* spp., to less well characterized heterotrophic anaerobic and aerobic bacteria.	29,541,788	29,975	974.93 - 1049.55
YLAKE: All Metagenomic 454 Shotgun Reads (N) (NCBI Bioproject PRJNA60433)	Yellowstone Lake is located in Yellowstone National Park (YNP), United States and is the largest (~352 km2) sub-alpine high-altitude lake in North America. It is a pristine, non-regulated body of water with a maximum measured depth of 131 m, an average depth of 42.5 m, and a long 10 yr water retention time. The lake's food web is critical to the function of the Yellowstone ecosystem, with the wellbeing of the park's many charismatic megafauna (grizzly bear, osprey, bald eagle, otter and many others) now recognized as being linked to the lake's productivity via the Yellowstone Cutthroat trout, a keystone species. Presumably, microbial food webs are foundational in this regard. A remotely operated vehicle was used for lake reconnaissance and for sampling of active hydrothermal vents. The habitats chosen included: i) near-surface (3m and 10 m depth) photic-zones; ii) high-temperature hydrothermal vents on the lake floor that contribute definable geochemical energy inputs; iii) microbial streamers; and iv) hydrothermal vent-water column mixing environments.	8,100,081,570	22,695,086	247.77 - 412.38

* NGS = Next Generation Sequencing
