# Supplementary material for: Bacterial Human Virulence Genes across Diverse Habitats As Assessed by In silico Analysis of Environmental Metagenomes
Source: Front Microbiol. 2016 Nov 3;7:1712. doi: 10.3389/fmicb.2016.01712 (PMC5093120; doi:10.3389/fmicb.2016.01712)
Supplement: Supplementary file 2 [file Table2.PDF]

Supplementary Table 2. Partial 16S/23S rRNA gene sequences used in BLASTN searches to test for the presence of clinically relevant bacteria in the metagenomes. The partial 16S/23S rRNA gene sequences are those normally amplified with species or group specific PCR primers.

Bacterium	rRNA gene	Sequence	Size (bp)	Reference strain	Primer reference
Escherichia coli	16S-23S	GTTAATGATAGTGTGTCGAAACACACTGGGTTTCCCCA<br>TTCGGAAATCGCCGGTTATAACGGTTCATATCACCTTA<br>CCGACGCTTATCGCAGATTAGCACGTCCTTCATCGCCT<br>CTGACTGCCAGGGCATCCACCGTGTACGCTTAGTCGCT<br>TAACCTCACAACCCGAAGATGTTTCACTTCAGAGTTGC<br>GAAAATTTGAGAGACTCACGAACAACTTTCATTGTTCA<br>GTGTTTCAATTTTCAGCTTGATCCAGATTTTTAAAGAG<br>CAAATATATCAAACAAGACTTAACAGTCTGTTTTGAGA<br>TATTGAGGTCGGCGACTTTCACTCACAAACCAGCAAGT<br>GGCGTCCCCTAGGGGATTCGAACCCCTGTTACCGCCGT<br>GAAAGGGCGGTGTCCTGGGCCTCTAGACGAAGGGGACA<br>CGAAAATTG	427	AE014075.1	1
Helicobacter pylori	16S	TCCCACACTCTAGAATAGTAGTCTCAAATGCAGTTCTA<br>TGGTTAAGCCATAGGATTTCACACCTGACTGACTATCC<br>CGCCTACGCGCTCTTTACGCCCAGTGATTCCGAGTAAC<br>GCTTGCACCCTCCGTATTACCGCGGCTGCTGGCACGGA<br>GTTAGCCGGTGCTTATTCGTTAGATACCGTCATTATCT<br>TCTCTAACAAAAGGAGTTTACAATCCTAAAACCTTCAT<br>CCTCCACGCGGCGTTGCTGCTTCAGGGTTTCCCCCATT<br>GAGCAATATTCCCTACTGCTGCCTCCCGTAGGAGTCTG<br>GACCGTGTCTCAGTTCCAGTGTGTCCGTTCACCCTCTC<br>AGGCCGGATACCCGTCATAGCCTTGGTAAGCCATTACC<br>TTACCAACAAGCTGATAGGACATAGGCTGATCTCTTAG<br>CGATAAATCTTTCCCCCGTAGGGAGTATCTGGTATTAA<br>TCATCGTTTCCAATGGCTATCCCAAACTAAGAGGCACA<br>TAACCTATGCGTTACTCACCCGTGCGCCACTAATCAGC<br>ACTCTAGCAAG	543	AP013356.1	2
Salmonella enterica	16S	ACGGTAACAGGAAGCAGCTTGCTGCTTTGCTGACGAGT<br>GGCGGACGGGTGAGTAATGTCTGGGAAACTGCCTGATG<br>GAGGGGGATAACTACTGGAAACGGTGGCTAATACCGCA<br>TAACGTCGCAAGACCAAAGAGGGGGACCTTCGGGCCTC<br>TTGCCATCAGATGTGCCCAGATGGGATTAGCTTGTTGG<br>TGAGGTAACGGCTCACCAAGGCGACGATCCCTAGCTGG<br>TCTGAGAGGATGACCAGCCACACTGGAACTGAGACACG<br>GTCCAGACTCCTACGGGAGGCAGCAGTGGGGAATATTG<br>CACAATGGGCGCAAGCCTGATGCAGCCATGCCGCGTGT<br>ATGAAGAAGGCCTTCGGGTTGTAAAGTACTTTCAGCGG<br>GGAGGAAGGTGTTGTGGTTAATA	403	NR_074934.1	3
Streptococcus pneumoniae	16S-23S	ATTGCCGAAGATTCCCTACTGCTGCCTCCCGTAGGAGT<br>CTGGGCCGTGTCTCAGTCCCAGTGTGGCCGATCACCCT<br>CTCAGGTCGGCTATGTATCGTCGCCTTGGTGAGCCCTT<br>ACCCCACCAACTAGCTAATACAACGCAGGTCCATCTGG<br>TAGTGATGCAAGTGCACCTTTTAAGCAAATGTCATGCA<br>ACATCCACTCTTATGCGGTATTAGCTATCGTTTCCAAT<br>AGTTATCCCCCGCTACCAGGCAGGTTACCTACGCGTTA<br>CTCACCCGTTCGCAACTCAT	286	CP006844.1	4
Staphylococcus aureus	16S	AATCTTTGTCGGTACACGATATTCTTCACGACTAAATA<br>AACGCTCATTCGCGATTTTATAAATGAATGTTGATAAC<br>AATGTTGTATTATCTACTGAAATCTCATTACG	108	CP007176.1	5

1. Khan,I.U.H. et al. (2007) Development of a rapid quantitative PCR assay for direct detection and quantification of culturable and non-culturable *Escherichia coli* from agriculture watersheds. *J. Microbiol. Methods* **69**, 480-488.
2. Thoreson,A.C.E. et al. (1995) Development of A PCR-Based Technique for Detection of *Helicobacter pylori*. *FEMS Immunol. Med. Microbiol.* **10**, 325-333.
3. Trkov,M. & Avgustin,G. (2003) An improved 16S rRNA based PCR method for the specific detection of *Salmonella enterica*. *Int. J. Food Microbiol.* **80**, 67-75.
4. Scholz,C.F., Poulsen,K., & Kilian,M. (2012) Novel molecular method for identification of *Streptococcus pneumoniae* applicable to clinical microbiology and 16S rRNA sequence-based microbiome studies. *J. Clin. Microbiol* **50**, 1968-1973.
5. Schmitz,F.J. et al. (1997) Specific information concerning taxonomy, pathogenicity and methicillin resistance of staphylococci obtained by a multiplex PCR. *J. Med. Microbiol* **46**, 773-778.
