# Supplementary material for: Bacterial Human Virulence Genes across Diverse Habitats As Assessed by In silico Analysis of Environmental Metagenomes
Source: Front Microbiol. 2016 Nov 3;7:1712. doi: 10.3389/fmicb.2016.01712 (PMC5093120; doi:10.3389/fmicb.2016.01712)
Supplement: Supplementary file 3 [file Table3.PDF]

Supplementary Table 3. Coverage, identity and E-value of best TBLASTN metagenomic hits to protein sequences of virulence genes. Best (in some cases also second best) NCBI BLASTP search results of metagenomic hits are also shown. Best matches not similar to expected virulence genes are shown in italics.

Metagenome	Coverage (%)	Identity (%)*	E-value	Best matches of NCBI BLAST of metagenomic hits
hlyA (1024 aa)				
Contaminated soil	6	48	8,19E-09	1) hypothetical protein ETSY1_39170 [Candidatus Entotheonella sp. TSY1] 2) haemolysin-type calcium-binding repeat [Methyloglobulus morosus KoM1]
DeepMed	7	49	8,13E-08	1) haemolysin-type calcium-binding repeat protein [Moorea producens 3L]
PacificOcean	7	44	1,85E-07	1) ApxIIA [Actinobacillus pleuropneumoniae] 2) RTX-II toxin determinant A [Actinobacillus pleuropneumoniae]
N. Pac. Line67	5	67	8,68E-08	1) alpha-haemolysin [Escherichia coli O75:H2]
PBSM	9	40	3,04E-08	1) Hypothetical protein [Methylobacterium mesophilicum]2) type I secretion protein [Thalassiobium sp. R2A62] >gb|EET49100.1| haemolysin-type calcium-binding region [Thalassiobium sp. R2A62]
GuaymasBasin	9	43	6,18E-07	1) hypothetical protein [Scytonema hofmanni]<br>2) Haemolysin-type calcium-binding region [Gloeocapsa sp. PCC 7428]
Bacterioplankton	6	58	8,94E-07	1) hypothetical protein, partial [Pseudomonas aeruginosa VRFPA07]<br>2) Haemolysin-related protein RbmC [Grimontia sp. AK16]
BATS	6	50	7,79E-07	1) hypothetical protein, partial [Legionella shakespearei]<br>2) Haemolysin-type calcium-binding region [Crocosphaera watsonii WH 8501]
BermudaOceanic	8	44	2,03E-07	1) Ca2+-binding protein, RTX toxin [Chamaesiphon minutus PCC 6605]
Drifting-ESP	6	48	4,82E-07	1) *type I secretion target repeat protein [Ketogulonicigenium vulgare Y25] >ref|YP_005796165.1| peptidyl-prolyl cis-trans isomerase cyclophilin type [Ketogulonicigenium vulgare WSH-001]*
HydrothermalVent	10	38	7,75E-07	1) Hypothetical protein [Methylobacterium mesophilicum]<br>2) haemolysin expression modulating protein [Skermanella stibiiresistens SB22]
	6	50	4,14E-09	1) Hypothetical protein [Methylobacterium mesophilicum]<br>2) Haemolysin-type calcium-binding region, RTX [Rhodobacter sphaeroides 2.4.1]
GutlessWorm	8	37	1,98E-10	1) VcbS [Simiduia agarivorans SA1 = DSM 21679]<br>2) Haemolysin-type calcium-binding region [Arthrospira maxima CS-328]
Hypersaline	11	44	1,16E-08	1) conserved repeat protein [Xenococcus sp. PCC 7305] 2) haemolysin, chromosomal, partial [Escherichia coli UMEA 3222-1]
WashingtonLake	17	35	4,63E-09	1) putative calcium-binding protein [Gloeocapsa sp. PCC 73106]<br>2) haemolysin-type calcium-binding protein [Paracoccus denitrificans PD1222]
MILOCO:454Shotgun	16	31	8,61E-08	1) putative lipoprotein [Glaciecola sp. HTCC2999]<br>2) haemolysin-type calcium-binding repeat (2 copies) [Rubidibacter lacunae]
HOT:Sanger	21	30	5,46E-10	1) periplasmic protein TonB links inner and outer membranes-like [Trichodesmium erythraeum IMS101]<br>2) haemolysin-type calcium-binding protein [Calothrix sp. PCC 6303]
	7	50	1,18E-07	1) hypothetical protein [Oscillatoria sp. PCC 10802]<br>2) Haemolysin-type calcium-binding region, RTX [Rhodobacter sphaeroides KD131]

Metagenome	Coverage (%)	Identity (%)*	E-value	Best matches of NCBI BLAST of metagenomic hits
YLAKE	10	44	3,25E-07	1) Haemolysin-type calcium-binding region [Oscillatoria nigro-viridis PCC 7112]
AntarcticaAquatic:454	9	46	2,16E-07	1) haemolysin-type calcium-binding protein [Paracoccus denitrificans PD1222]
BotanyBay:454	10	50	4,12E-09	1) Hypothetical protein [Methylobacterium mesophilicum]<br>2) haemolysin-type calcium-binding protein [Paracoccus denitrificans PD1222]
AntarcticaAquatic:Sanger	9	52	3,31E-08	1) hypothetical protein [Loktanella vestfoldensis]<br>2) haemolysin expression modulating protein [Microvirga lotononidis]
BisonMetagenome	8	50	6,39E-10	1) haemolysin-type calcium-binding protein [Candidatus Chloracidobacterium thermophilum B]
GOS	12	52	1,78E-10	1) bifunctional haemolysin-adenylate cyclase precursor [Vibrio caribbenthicus]
MILOCO	9	46	1,30E-07	1) Hypothetical protein [Methylobacterium mesophilicum]<br>2) haemolysin-type calcium-binding region [Paracoccus aminophilus JCM 7686]
hylA (1066 aa)				
MILOCO:454Shotgun	14	38	4,31E-18	1) hypothetical protein [Virgibacillus halodenitrificans]<br>2) hyaluronidase, partial [Bacillus sp. A50]
WashingtonLake	28	31	6,65E-37	1) Hyaluronate lyase [Paenibacillus alvei TS-15]
YLAKE	15	47	1,29E-21	1) hypothetical protein [Paenibacillus terrigena]<br>2) hyaluronidase, partial [Bacillus sp. A50]
AntarcticaAquatic:454	13	33	1,62E-11	1) Select seq ref|WP_009134339.1| hypothetical protein [Alistipes indistinctus YIT 12060]<br>2) hyaluronidase, partial [Bacillus sp. A50]
GOS	28	33	1,15E-36	1) hyaluronate lyase [Paenibacillus alvei TS-15]
papH (195 aa)				
AntarcticaAquatic:Sanger	76	34	2,75E-16	1) pilin [Yokenella regensburgei] >gb|EHM45805.1| fimbrial protein [Yokenella regensburgei ATCC 43003]
papA (199 aa)				
GutlessWorm	80	34	1,12E-08	1) putative fimbrial protein [Pseudomonas sp. FH4]
BisonMetagenom	61	36	9,28E-09	1) fimbrial protein [Enterobacter cloacae complex]
GOS	99	35	8,12E-15	1) type 1 fimbriae major subunit FimA [Burkholderia vietnamiensis]
MILOCO:454	85	31	1,05E-09	1) Select seq ref|WP_023659208.1| P pilus assembly protein, pilin FimA [Pseudomonas fluorescens]
papC (839 aa)				
SalternMetagenome	4	74	3,17E-08	1) P pilus assembly protein, porin PapC [Serratia marcescens VGH107]
BATS	10	44	1,09E-08	1) outer membrane usher protein htrE domain protein [Shigella boydii 5216-82]
CellCapture	10	44	5,35E-11	1) fimbrial biogenesis outer membrane usher protein [Pseudomonas sp. CFT9]
Drifting-ESP	6	49	5,65E-07	1) papC N-terminal domain protein [Escherichia coli]
TermiteGut	24	33	3,55E-18	1) fimbrial Usher family protein [Shigella sonnei str. Moseley]
IceMetagenome	10	42	2,41E-11	1) fimbrial Usher family protein [Shigella sonnei 3233-85]

Metagenome	Coverage (%)	Identity (%)*	E-value	Best matches of NCBI BLAST of metagenomic hits
WesternChannelOMM	18	51	1,36E-42	1) hypothetical protein [Escherichia coli] >gb|EQX73347.1| 2) outer membrane usher papC domain protein [Escherichia coli EPECa14]
YLAKE	17	39	1,11E-16	1) outer membrane usher protein FimD [Morganella sp. EGD-HP17]
AntarcticaAquatic:454	19	39	2,22E-19	1) fimbrial protein [Xylella fastidiosa PLS229]
BotanyBay:454	17	41	1,14E-21	1) fimbrial usher protein [Enterobacter sp. MGH 13]
	9	64	7,29E-20	1) Select seq ref|WP_023563779.1| fimbrial biogenesis outer membrane usher protein, partial [Escherichia coli]
BisonMetagenome	98	34	2,22E-14	
	31	51	1,13E-65	1) fimbrial usher protein [Enterobacter sp. MGH 13]
GOS	79	41	3,89E-74	
	38	47	1,30E-82	1) outer membrane usher protein [Aeromonas hydrophila ML09-119]
MILOCO:454	80	42	4,41E-11	
	15	62	8,62E-38	1) fimbrial protein SteB [Pseudomonas sp. Ag1]
focG (167 aa)				
BisonMetagenome	99	32	5,69E-09	1) Select seq dbj|BAO33011.1| fimbrial subunit [Serratia marcescens SM39]
GOS	98	45	8,92E-23	1) type 1 fimbrae adaptor subunit FimG [Raoultella ornithinolytica B6]
MILOCO:454	67	43	6,79E-16	1) fimbrial protein FimG [Pseudomonas fluorescens]
fimH (303 aa)				
Drifting-ESP	17	63	2,30E-07	1) mannose-binding domain protein FimH [Escherichia coli 2.4168]
IceMetagenome	23	99	4,50E-33	1) Select seq ref|WP_023154120.1| protein FimH [Escherichia coli]
GOS	25	81	1,91E-24	1) Select seq ref|WP_002916197.1| fimbrial protein FimH [Klebsiella pneumoniae]
MILOCO:454	55	51	4,56E-40	1) fimbrial protein FimH [Pseudomonas fluorescens BBc6R8]
	38	64	1,06E-36	1) fimbrial protein FimH [Pseudomonas fluorescens BBc6R8]
	12	81	2,83E-09	1) Select seq dbj|BAO33011.1| fimbrial subunit [Serratia marcescens SM39]
invA (685 aa)				
Microbialites	5	79	7,33E-08	1) flagellar biosynthesis protein FlhA [Hirschia maritima]
PeruMarginSediment	4	77	9,42E-07	1) flagellar biosynthesis protein flhA domain protein [Shigella dysenteriae 155-74]
Mountain lake	9	65	1,71E-16	1) hypothetical protein [Limnohabitans sp. Rim28]<br>2) flagellar biosynthesis protein FlhA [gamma proteobacterium HIMB30]
GeneExpression	12	52	7,55E-14	1) flagellar biosynthesis protein FlhA [Thioalkalivibrio sp. ALJ17]
	6	68	2,35E-09	1) flagellar biosynthesis protein FlhA [Thioalkalivibrio sp. ALJ17]
AmazonRiverPlume	13	53	8,90E-15	1) flagellar biosynthesis protein FlhA [Rhodobacteraceae bacterium HIMB11]
	6	68	1,30E-07	1) flagellar biosynthesis protein FlhA [Rhodobacteraceae bacterium HIMB11]
Sapelo2008	12	61	7,30E-15	1) flagellar biosynthesis protein FlhA [Paenibacillus daejeonensis]
	6	73	9,44E-18	1) flagellar biosynthesis protein FlhA [Desulfotalea psychrophila LSv54]
SargassoSea	12	53	3,25E-13	1) hypothetical protein [alpha proteobacterium SCGC AAA158-B04]<br>2) flagellar biosynthesis protein FlhA [SAR116 cluster alpha proteobacterium HIMB100]
	6	71	2,01E-08	1) flagellar biosynthesis protein FlhA [Thalassolituus oleivorans MIL-1]

Metagenome	Coverage (%)	Identity (%)*	E-value	Best matches of NCBI BLAST of metagenomic hits
AcidMine	36	49	1,45E-45	1) flagellar biosynthetic protein FlhA [Leptospirillum ferriphilum ML-04]
	94	34	4,93E-31	1) flagellar biosynthetic protein FlhA [Leptospirillum ferriphilum ML-04]
MontereyBay transect	8	70	2,63E-15	1) flagellar biosynthesis protein FlhA [gamma proteobacterium HTCC2207]
PacificOcean	17	57	6,16E-16	1) flagellar biosynthesis protein FlhA [Candidatus Puniceispirillum marinum IMCC1322]
	6	71	1,25E-08	1) flagellar biosynthesis protein FlhA [alpha proteobacterium SCGC AAA536-K22]
HOT:454	13	61	1,95E-17	1) hypothetical protein, partial [Nitrospina sp. AB-629-B06]<br>2) flagellar biosynthesis protein FlhA [Sulfurihydrogenibium azorense Az-Fu1]
	8	71	1,47E-16	1) type III secretion integral inner membrane protein [Simkania negevensis Z]
MontereyBay	12	60	1,97E-13	1) Flagellar biosynthesis protein FlhA [Gammaproteobacteria bacterium MOLA455]
	9	65	1,24E-16	1) flagellar biosynthesis protein FlhA [Octadecabacter antarcticus 307]
N. Pac. Line67	18	60	2,35E-25	1) Flagellar biosynthesis protein FlhA, partial [Salmonella enterica subsp. enterica serovar Rubislaw str. A4-653]
	7	72	3,38E-12	1) flagellar biosynthesis protein FlhA [Bradyrhizobium diazoefficiens USDA 110]
PBSM	24	57	7,93E-39	1) *flagella-associated protein [Bdellovibrio bacteriovorus str. Tiberius]*
SalternMetagenome	6	68	7,85E-08	1) type III secretion system protein InvA, partial [Salmonella enterica subsp. enterica serovar London str. CFSAN001081]
AlvinellaPompejana	24	46	3,57E-07	1) flagellar biosynthesis protein FlhA [Spirochaeta smaragdinae DSM 11293]
GuaymasBasin	24	54	3,08E-29	1) flagellar biosynthesis protein FlhA [Alteromonas macleodii str. 'Black Sea 11']
	6	80	6,73E-11	1) flagellar biosynthesis protein FlhA [Paenibacillus senegalensis]
BotanyBay:Sanger	34	50	1,29E-42	1) type III secretion FHIPEP protein [Turneriella parva DSM 21527]
Bacterioplankton	12	65	3,27E-16	1) hypothetical protein [alpha proteobacterium SCGC AAA158-B04]<br>2) predicted flagellar biosynthesis protein FlhA [uncultured organism MedDCM-OCT-S09-C206]
	7	80	5,45E-11	1) InvA [Salmonella enterica subsp. enterica serovar Abony]
BATS	13	62	8,44E-16	1) Select seq ref|WP_010491278.1| flagellar biosynthesis protein FlhA [Paenibacillus elgii]
	7	70	6,48E-07	1) flagellar biosynthesis protein FlhA [Desulfocapsa sulfexigens DSM 10523]
BermudaOceanic	26	54	7,95E-32	1) flagellar biosynthesis protein FlhA [Sporolactobacillus laevolacticus]
	9	68	2,77E-18	1) flagellar biosynthesis protein FlhA [Sulfuricella denitrificans skB26]
CellCapture	12	70	1,79E-15	1) type III secretion protein rscV [Pseudomonas fluorescens WH6]
	9	75	1,09E-18	
Drifting-ESP	23	47	2,80E-24	1) flagellar biosynthesis protein FlhA [Oceanibaculum indicum P24]
	6	64	9,43E-16	1) flagellar biosynthesis protein FlhA [Oceanibaculum indicum P24]
HydrothermalVent	35	54	1,89E-50	1) flagellar biosynthesis protein FlhA [Thiomicrospira crunogena XCL-2]
TermiteGut	76	34	1,29E-55	1) flagellar biosynthesis protein FlhA [Treponema caldaria DSM 7334]
	31	55	1,45E-08	1) hypothetical protein, partial [Bacillus sp. EGD-AK10]<br>2) Flagellar biosynthesis protein FlhA, partial [Candidatus Arthromitus sp.

Metagenome	Coverage (%)	Identity (%)*	E-value	Best matches of NCBI BLAST of metagenomic hits
				SFB-4]
GutlessWorm	39	48	1,85E-46	1) flagellar biosynthesis protein FlhA [Desulfarculus baarsii DSM 2075]
HypersalineMat	31	50	2,34E-35	1) flagellar biosynthesis protein FlhA [Pannonibacter phragmitetus]
IceMetagenome	12	64	1,98E-15	1) flagellar biosynthesis protein FlhA [Austwickia chelonae] >dbj|GAB77775.1|
	9	71	2,64E-18	1) type III secretory pathway, component EscV [Candidatus Chloracidobacterium thermophilum B]
WesternChannelOMM	21	57	1,29E-22	1) Select seq ref|WP_007153960.1| flagellar biosynthesis protein FlhA [Marinobacter algicola DG893]
	6	71	2,24E-20	1) flagellar biosynthesis protein FlhA [gamma proteobacterium HIMB30]
WashintonLake	95	33	3,23E-68	1) flagellar biosynthesis protein FlhA [Methylotenera mobilis JLW8]
	35	53	3,27E-33	1) flagellar biosynthesis pathway, FHIPEP (flagella/HR/invasion proteins export pore) family [Ralstonia syzygii R24]<br>2) type III secretion FHIPEP [Shewanella denitrificans OS217] 75% identity
MILOCO:454Shotgun	24	65	1,70E-33	1) Select seq ref|WP_009819757.1| type III secretion protein [Roseovarius sp. 217]
	9	75	2,84E-18	1) Type III secretory pathway, component EscV [uncultured bacterium]
HOT:Sanger	34	53	1,56E-14	1) hypothetical protein [alpha proteobacterium SCGC AAA158-B04]<br>2) flagellar biosynthesis protein FlhA [Loktanella vestfoldensis SKA53]
	9	65	5,03E-15	1) flagellar biosynthesis protein FlhA [Bacillus methanolicus PB1]
YLAKE	25	59	1,32E-33	1) flagellar biosynthesis protein FlhA [Sulfurihydrogenibium yellowstonense SS-5]
	5	78	4,24E-07	1) flagellar biosynthesis protein FlhA [gamma proteobacterium HTCC2207
AntarcticaAquatic:454	27	67	1,25E-13	1) flagellar biosynthesis protein [Halobacillus halophilus DSM 2266]
	7	75	2,26E-13	1) flagellar biosynthesis protein FlhA [Clostridium hathewayi] >gb|ENY90719.1|
BotanyBay:454	26	51	4,73E-29	1) flagellar biosynthesis protein FlhA [Verrucomicrobiae bacterium DG1235]
	18	70	3,88E-17	1) Type III secretion inner membrane channel protein (LcrD,HrcV,EscV,SsaV [Achromobacter xylosoxidans NH44784-1996]
AntarcticaAquatic:Sanger	36	56	1,73E-29	1) Select seq gb|EPP37082.1| FHIPEP family protein, partial [Chlamydia psittaci 84-8471/1] 73% identity<br>2) type III secretion inner membrane protein SctV [Chlamydia muridarum str. Nigg CM972] 73% identity
	47	43	2,73E-46	1) type III secretion pathway protein SctV [Candidatus Protochlamydia amoebophila UWE25]
BisonMetagenome	54	39	3,27E-33	1) hypothetical protein [Fervidibacteria bacterium SCGC AAA471-D06]<br>2) Select seq ref|YP_006439067.1| type III secretion FHIPEP protein [Turneriella parva DSM 21527]
	40	54	1,92E-30	1) flagellar biosynthesis protein FlhA [Chthonomonas calidirosea T49]
GOS	39	61	3,73E-19	1) Type III secretion inner membrane channel protein (LcrD,HrcV,EscV,SsaV) [Thiorhodococcus sp. AK35]
	9	77	6,00E-18	
MILOCO:454	23	63	2,54E-28	1) type III secretion protein [Pseudomonas fluorescens BBc6R8]
	5	81	5,10E-07	

Metagenome	Coverage (%)	Identity (%)*	E-value	Best matches of NCBI BLAST of metagenomic hits
spiA (497 aa)				
AlvinellaPompejana	30	30	7,83E-08	1) general secretion pathway protein GspD [Sulfurovum sp. AS07-7]
BotanyBay:Sanger	42	31	1,46E-12	1) general secretion pathway protein D [gamma proteobacterium IMCC3088]
Bacterioplankton	17	38	1,44E-08	1) hypothetical protein [Sphingomonas-like bacterium B12]<br>2) putative type III secretion protein YscC [Sphingomonas sp. SKA58]
CellCapture	17	33	7,48E-07	1) secretin [Pseudomonas extremaustralis]
WesternChannelOMM	18	38	9,24E-07	1) *Type IV pilus biogenesis and competence protein PilQ precursor [Gammaproteobacteria bacterium MOLA455]*
WashintonLake	14	40	6,57E-21	1) type II secretion system protein GspD [Oceanospirillum beijerinckii]
MILOCO:454Shotgun	30	34	7,31E-12	1) *Type IV pilus biogenesis and competence protein PilQ precursor [Gammaproteobacteria bacterium MOLA455]*
	30	31	3,50E-15	1) secretory protein (associated with virulence) [Salmonella enterica subsp. enterica serovar Paratyphi A str. ATCC 9150]
HOT:Sanger	22	32	4,77E-09	1) type II and III secretion system protein [Fibrobacter succinogenes]
YLAKE	29	30	1,34E-07	1) type II and III secretion system protein [Pedosphaera parvula]
AntarcticaAquatic:454	34	31	8,79E-10	1) *type 4 fimbrial biogenesis protein PilQ [Reinekea sp. MED297]*
	32	31	1,89E-08	1) type III secretion outer membrane pore, YscC/HrcC family [Herbaspirillum sp. YR522]
BotanyBay:454	36	34	9,08E-17	1) putative type III secretion protein YscC [Hahella chejuensis KCTC 2396]
AntarcticaAquatic:Sanger	16	35	2,32E-07	1) fimbrial protein [Gammaproteobacteria bacterium MOLA455]
BisonMetagenome	36	39	1,44E-08	1) type II and III secretion system protein [Sporomusa ovata DSM 2662]
GOS	54	36	2,22E-39	1) Select seq ref|WP_007175539.1| type III secretion protein [Burkholderia sp. Ch1-1]
MILOCO:454	33	36	3,65E-19	1) secretin [Pseudomonas sp. PAMC 25886]
phoP (224 aa)				
LineIsland	17	63	1,87E-07	1) Select seq ref|WP_009723795.1| transcriptional regulator [Halomonas sp. TD01]
Mountain lake	30	45	7,45E-09	1) two component transcriptional regulator [Verminephrobacter eiseniae EF01-2]
GeneExpression	30	45	3,98E-21	1) phosphate regulon transcriptional regulatory protein PhoB [Variovorax paradoxus B4]
	23	65	3,38E-12	1) transcriptional regulator [Sphingomonas sp. Mn802worker]
Contaminated soil	57	41	3,89E-23	1) transcriptional regulator [Hyphomicrobium zavarzinii]
AmazonRiverPlume	40	58	2,07E-22	1) transcriptional regulator [Hyphomicrobium zavarzinii]
DeepMed	97	33	8,46E-25	1) ArsR family transcriptional regulator [Pedosphaera parvula] >gb|EEF59372.1| two component transcriptional regulator, winged helix family [bacterium Ellin514]
Sapelo2008	41	56	1,17E-20	1) transcriptional regulator [marine gamma proteobacterium HTCC2143] >gb|EAW31431.1| two-component system regulatory protein [marine gamma proteobacterium HTCC2143]
SargassoSea	34	53	1,70E-13	1) two-component system response regulator [Synechococcus sp. RCC307]
Yellowstone	99	34	5,18E-29	1) DNA-binding response regulator [Synechococcus sp. JA-3-3Ab] >ref|WP_011431117.1| transcriptional regulator [Synechococcus sp. JA-3-3Ab]
AcidMine	97	42	1,63E-32	1) two component transcriptional regulator [Petrotoga mobilis SJ95]

Metagenome	Coverage (%)	Identity (%)*	E-value	Best matches of NCBI BLAST of metagenomic hits
				>ref|WP_012208678.1| PhoB family transcriptional regulator [Petrotoga mobilis] >gb|ABX31575.1| two component transcriptional regulator, winged helix family [Petrotoga mobilis SJ95]
MontereyBay transect	47	35	9,42E-08	1) chemotaxis protein CheY [Candidatus Pelagibacter ubique]
	33	47	3,54E-14	1) two-component response regulator [Prochlorococcus marinus subsp. pastoris str. CCMP1986] >ref|WP_011131767.1| chemotaxis protein CheY [Prochlorococcus marinus]
PacificOcean	67	36	5,93E-17	1) chemotaxis protein CheY [Crocosphaera watsonii] >gb|EHJ14797.1| Two component Transcriptional regulator, Winged helix family protein [Crocosphaera watsonii WH 0003]
	23	63	1,46E-12	1) Select seq ref|WP_006853969.1| chemotaxis protein CheY [Synechococcus sp. WH 8016] >gb|EHA61762.1| two component transcriptional regulator, winged helix family [Synechococcus sp. WH 8016]
HOT:454	37	51	2,08E-20	1) Select seq ref|WP_022698693.1| transcriptional regulator [Maricaulis sp. JL2009]
MontereyBay	47	35	1,04E-07	1) chemotaxis protein CheY [Candidatus Pelagibacter ubique]
	33	54	2,80E-17	1) transcriptional regulator [marine gamma proteobacterium HTCC2143] >gb|EAW31431.1| two-component system regulatory protein [marine gamma proteobacterium HTCC2143]
N. Pac. Line67	58	46	3,04E-27	1) response regulator in two-component regulatory system with PhoQ [Pseudoalteromonas agarivorans S816]
	21	67	2,30E-22	
PBSM	97	35	1,87E-25	1) hypothetical protein [Aminicenantes bacterium SCGC AAA252-O11]<br>2) Select seq ref|WP_007414271.1| two component transcriptional regulator, winged helix family [Pedosphaera parvula]
SalternMetagenome	15	94	1,32E-11	1) Transcriptional regulatory protein PhoP [Klebsiella pneumoniae IS10]
AlvinellaPompejana	90	33	4,36E-20	1) two-component response regulator [Sulfurovum sp. NBC37-1] >ref|WP_012084051.1| chemotaxis protein CheY [Sulfurovum sp. NBC37-1]
GuaymasBasin	72	42	1,01E-35	1) response regulator consisting of a CheY-like receiver domain and a winged-helix DNA-binding domain-containing protein [Cycloclasticus sp. P1]
BotanyBay:Sanger	99	47	7,69E-48	1) transcriptional regulator [Amphritea japonica]
Bacterioplankton	38	48	5,25E-16	1) two-component response regulator [Prochlorococcus marinus str. MIT 9515] >ref|WP_011819480.1| chemotaxis protein CheY [Prochlorococcus marinus]
BATS	33	57	5,89E-17	1) transcriptional regulator [Methylophaga aminisulfidivorans] >gb|EGL55111.1| response regulator consisting of a CheY-like receiver domain and a winged-helix DNA-binding domain containing protein [Methylophaga aminisulfidivorans MP]
BermudaOceanic	78	35	9,66E-22	1) PhoB family transcriptional regulator [Candidatus Pelagibacter sp. HTCC7211]
CellCapture	34	56	3,50E-19	1) transcriptional regulator [Pseudomonas] >gb|EJF70736.1| two-component system transcriptional regulatory protein PhoP [Pseudomonas sp. Ag1] >gb|ESW54596.1| Response regulator with CheY and a winged-helix DNA-binding domain [Pseudomonas fluorescens BBc6R8]
Drifting-ESP	49	51	1,00E-19	1) transcriptional regulator [marine gamma proteobacterium HTCC2143] >gb|EAW31431.1| two-component system regulatory protein [marine gamma proteobacterium HTCC2143]
HydrothermalVent	97	35	2,87E-28	1) two component transcriptional regulator [Thiomicrospira crunogena XCL-2] >ref|WP_011369962.1| chemotaxis protein CheY [Thiomicrospira crunogena]

Metagenome	Coverage (%)	Identity (%)*	E-value	Best matches of NCBI BLAST of metagenomic hits
TermiteGut	95	36	1,89E-28	1) response regulator with CheY-like receiver domain and winged-helix DNA-binding domain [Desulfosporosinus orientis DSM 765] >ref|WP_014186318.1| PhoB family transcriptional regulator [Desulfosporosinus orientis]
GutlessWorm	99	40	1,96E-39	1) two-component system regulatory protein [Methylophaga lonarensis MPL]
HypersalineMat	97	36	3,90E-25	
	78	41	3,93E-32	1) Two-component cell cycle transcriptional regulator ctrA [Azospirillum lipoferum 4B]
IceMetagenome	45	56	7,76E-20	1) Response regulator receiver domain [uncultured organism]
	17	64	2,29E-12	
WesternChannelOMM	74	63	2,51E-45	1) virulence transcriptional regulatory protein phoP [Escherichia coli UMEA 3656-1]
	48	92	1,39E-51	1) DNA-binding protein [Escherichia coli] >gb|EGX11649.1| transcriptional regulatory protein phoP [Escherichia coli G58-1]
WashintonLake	99	42	2,44E-43	1) transcriptional regulator [Methylobacter tundripaludum] >gb|EGW20695.1| two component transcriptional regulator, winged helix family [Methylobacter tundripaludum SV96]
MILOCO:454Shotgun	77	49	2,45E-39	1) PhoB family transcriptional regulator [Teredinibacter turnerae T7901] >ref|WP_015820189.1| transcriptional regulator [Teredinibacter turnerae] >gb|ACR14073.1| transcriptional regulatory protein PhoP [Teredinibacter turnerae T7901]
	25	62	6,40E-11	
HOT:Sanger	94	43	3,14E-41	1) response regulator in two-component regulatory system with PhoQ, regulates gene expression at low (Mg+) (OmpR family) [Candidatus Contendobacter odensis Run_B_J11]
YLAKE	71	44	7,47E-34	1) Select seq ref|WP_008210636.1| transcriptional regulator [Rhodanobacter sp. 115] >gb|EIL96800.1| response regulator with CheY-like receiver domain and winged-helix DNA-binding domain [Rhodanobacter sp. 115]
	16	61	6,72E-11	
AntarcticaAquatic:454	71	51	2,56E-36	1) response regulator in two-component regulatory system with PhoQ [Alteromonas sp. SN2]
	17	68	3,76E-08	
BotanyBay:454	79	48	6,86E-37	1) transcriptional regulator [Microbulbifer variabilis]
	20	68	3,10E-10	
AntarcticaAquatic:Sanger	47	85	5,92E-40	1) transcriptional regulator [Rahnella sp. Y9602] >ref|YP_005200916.1| response regulator with CheY-like receiver domain and winged-helix DNA-binding domain [Rahnella aquatilis CIP 78.65 = ATCC 33071] >ref|YP_005402734.1| DNA-binding transcriptional regulator PhoP [Rahnella aquatilis HX2]
	99	33	1,41E-29	1) alkaline phosphatase [Candidatus Aquiluna sp. IMCC13023] >gb|EIC92415.1| putative two component system response regulator [Candidatus Aquiluna sp. IMCC13023]
BisonMetagenome	99	94	6,29E-114	1) PhoP family transcriptional regulator [Enterobacter cloacae]
GOS	84	91	9,9858E-89	1) DNA-binding transcriptional regulator PhoP [Raoultella ornithinolytica B6] >ref|WP_004860631.1| PhoP family transcriptional regulator [Enterobacteriaceae] >gb|EHT10111.1| virulence transcriptional regulatory protein phoP [Klebsiella oxytoca 10-5246] >gb|AGJ86480.1| DNA-binding transcriptional regulator PhoP [Raoultella ornithinolytica B6]
MILOCO:454	75	39	8,99E-24	1) transcriptional regulator [Pseudomonas] >gb|EJF68812.1| two component system, response regulator [Pseudomonas sp. Ag1] >gb|ESW56047.1| Response regulator with CheY and a winged-helix DNA-binding domain

Metagenome	Coverage (%)	Identity (%)*	E-value	Best matches of NCBI BLAST of metagenomic hits
				[Pseudomonas fluorescens BBc6R8]
	36	97	1,48E-30	1) transcriptional regulator [Escherichia coli] >gb|EFF02096.1| two component transcriptional regulator [Escherichia coli FVEC1412] >gb|EGI46909.1| transcriptional regulatory protein PhoP [Escherichia coli H591]
phoQ (487 aa)				
FLAS	21	32	2,32E-09	1) Select seq ref|YP_444282.1| light- and oxygen-sensing transcription regulator [Salinibacter ruber DSM 13855]
AmazonRiverPlume	14	37	8,43E-07	1) signal transduction histidine kinase [SAR116 cluster alpha proteobacterium HIMB100]
Sapelo2008	15	45	2,32E-10	1) Select seq ref|WP_007021850.1| Sensory transduction histidine kinase [Neptuniibacter caesariensis] >gb|EAR62832.1| Sensory transduction histidine kinase [Oceanospirillum sp. MED92]
Yellowstone	20	35	3,04E-08	1) two-component sensor histidine kinase [Synechococcus sp. JA-2-3B'a(2-13)]
AcidMine	25	31	1,09E-08	1) sensor protein PhoQ, partial [Salmonella enterica subsp. enterica serovar Heidelberg str. N653]
MontereyBay transect	13	50	2,86E-12	1) sensor protein PhoQ [Plesiomonas shigelloides 302-73]
PacificOcean	13	46	2,74E-08	1) sensor protein PhoQ [Phtorhabdus temperata J3]
HOT:454	10	94	3,44E-18	1) histidine kinase [Escherichia sp. TW09308]
MontereyBay	18	42	6,70E-14	1) Sensor protein PhoQ [Methylophaga nitratireducenticrescens]
SalternMetagenome	7	77	6,02E-11	1) putative truncated sensory histidine kinase in two-component regulatory system with PhoP [Serratia symbiotica str. Tucson]
GuaymasBasin	29	37	2,00E-17	1) Integral membrane sensor signal transduction histidine kinase [Methylomicrobium alcaliphilum 20Z]
BotanyBay:Sanger	58	36	7,61E-39	1) Sensory transduction histidine kinase [Neptuniibacter caesariensis] >gb|EAR62832.1| Sensory transduction histidine kinase [Oceanospirillum sp. MED92]
Bacterioplankton	19	37	2,78E-07	1) histidine kinase with HAMP domain protein [alpha proteobacterium HIMB5]
BATS	14	50	1,06E-11	1) sensor protein PhoQ [Dickeya dadantii Ech703] >ref|WP_015853764.1| sensor protein PhoQ [Dickeya] >gb|ACS85855.1| histidine kinase [Dickeya dadantii Ech703]
BermudaOceanic	37	32	5,47E-14	1) histidine kinase [Pseudochrobactrum sp. AO18b]
CellCapture	18	86	1,42E-39	1) sensor protein PhoQ, partial [Escherichia coli]
Drifting-ESP	21	51	1,64E-21	1) Sensory transduction histidine kinase [Neptuniibacter caesariensis] >gb|EAR62832.1| Sensory transduction histidine kinase [Oceanospirillum sp. MED92]
HydrothermalVent	25	31	2,05E-12	1) two-component sensor histidine kinase [Thioclava sp. 13D2W-2]
TermiteGut	20	35	3,04E-08	1) PAS domain-containing sensor histidine kinase [Synechococcus sp. JA-2-3B'a(2-13)]
GutlessWorm	33	33	5,57E-16	1) histidine kinase [Thiorhodospira sibirica]
HypersalineMat	28	36	2,39E-10	1) PAS domain-containing sensor histidine kinase [Desulfomonile tiedjei]
IceMetagenome	15	74	5,32E-27	1) sensor protein PhoQ, partial [Escherichia coli]
WesternChannelOMM	31	79	1,12E-69	1) sensor protein PhoQ, partial [Escherichia coli]
	10	92	3,80E-44	

**Metagenome**	**Coverage (%)**	**Identity (%)***	**E-value**	**Best matches of NCBI BLAST of metagenomic hits**
WashintonLake	55	31	1,65E-27	1) histidine kinase [Methylotenera mobilis JLW8]
MILOCO:454Shotgun	36	43	1,44E-29	1) sensor protein PhoQ [Klebsiella oxytoca]
HOT:Sanger	48	35	9,57E-18	1) histidine kinase [sulfur-oxidizing symbionts] >gb|EGV51451.1| peptidase, M48 family [endosymbiont of Riftia pachyptila (vent Ph05)] >gb|EGW53358.1| sensor protein PhoQ [endosymbiont of Tevnia jerichonana (vent Tica)]
YLAKE	38	39	3,16E-18	1) histidine kinase [Blastomonas sp. CACIA14H2]
	28	46	2,27E-15	
AntarcticaAquatic:454	29	41	1,34E-26	1) integral membrane sensor signal transduction histidine kinase [Glaciecola sp. 4H-3-7+YE-5]
BotanyBay:454	55	40	9,12E-20	1) histidine kinase [Cellvibrio sp. BR] >gb|EIK43778.1| Sensory transduction histidine kinase [Cellvibrio sp. BR]
	10	81	8,19E-15	
AntarcticaAquatic:Sanger	37	51	4,20E-48	1) signal transduction histidine kinase [Rahnella aquatilis CIP 78.65 = ATCC 33071] >ref|WP_015697910.1| sensor protein PhoQ [Rahnella aquatilis] >gb|AEX52777.1| signal transduction histidine kinase [Rahnella aquatilis CIP 78.65 = ATCC 33071]
BisonMetagenome	53	82	2,12E-109	1) sensor protein phoQ [Enterobacter cloacae UCICRE 9]
GOS	69	78	2,57E-144	1) sensor protein PhoQ [Raoultella ornithinolytica B6] >ref|WP_004860627.1| sensor protein PhoQ [Enterobacteriaceae] >gb|EHT10110.1| sensor protein phoQ [Klebsiella oxytoca 10-5246] >gb|AGJ86481.1|
MILOCO:454	26	88	8,20E-62	1) sensor protein phoQ [Clonorchis sinensis]
rfbE (364 aa)				
FLAS	39	34	3,22E-15	1) aminotransferase DegT, partial [Curtobacterium sp. B18]
LineIsland	10	65	6,97E-08	1) perosamine synthetase [Escherichia coli DEC7E]
PeruMarginSediment	10	61	6,01E-08	1) glutamine--scyllo-inositol transaminase [Rhizobium leguminosarum bv. trifolii WSM2304]
Mountain lake	20	60	1,08E-19	1) DegT/DnrJ/EryC1/StrS aminotransferase family protein [Enhydrobacter aerosaccus SK60]
GeneExpression	23	51	1,24E-15	1) DegT/DnrJ/EryC1/StrS family protein [Thioalkalivibrio nitratireducens DSM 14787]
Contaminated soil	15	45	5,14E-14	1) *Pleiotropic regulatory protein [Flavobacterium sp. WG21]*
AmazonRiverPlume	24	55	2,01E-17	1) *pleiotropic regulatory protein DegT [Fusobacterium nucleatum subsp. animalis ATCC 51191]*
	10	66	4,72E-09	1) perosamine synthetase [Methanothermobacter thermautotrophicus str. Delta H] >ref|WP_010875973.1| aminotransferase DegT [Methanothermobacter thermautotrophicus] >gb|AAB84840.1| perosamine synthetase [Methanothermobacter thermautotrophicus str. Delta H]
DeepMed	78	32	2,61E-36	1) DegT/DnrJ/EryC1/StrS aminotransferase [Candidatus Desulforudis audaxviator MP104C]
Sapelo2008	22	69	4,56E-20	1) glutamate--GDP-4-keto-6-deoxy-D-mannose aminotransferase [Geobacter bemidjiensis Bem] 67% identity<br>2) perosamine synthase, partial [Escherichia coli TW14313]
SargassoSea	21	52	6,91E-18	1) Bacillosamine/Legionaminic acid biosynthesis aminotransferase PglE; 4-keto-6-deoxy-N-Acetyl-D-hexosaminyl-(Lipid carrier) aminotransferase [Dehalobacter sp. DCA]
Yellowstone	81	36	1,17E-41	1) DegT/DnrJ/EryC1/StrS aminotransferase family protein [Synechococcus sp. JA-3-3Ab]
AcidMine	77	49	4,36E-75	1) hypothetical protein [Ferroplasma sp. Type II] 2) aminotransferase DegT

Metagenome	Coverage (%)	Identity (%)*	E-value	Best matches of NCBI BLAST of metagenomic hits
				[Methanothermobacter thermautotrophicus] >gb|AAB84840.1| perosamine synthetase [Methanothermobacter thermautotrophicus str. Delta H]
	12	63	4,72E-09	
MontereyBay transect	26	57	6,12E-19	1) DegT/DnrH/EryC1/StrS aminotransferase family protein [alpha proteobacterium HIMB5]
	13	69	3,91E-10	1) 4-keto-6-deoxy-N-Acetyl-D-hexosaminyl-(Lipid carrier) aminotransferase [Campylobacter concisus ATCC 51562]
PacificOcean	37	41	1,36E-22	1) DegT/DnrJ/EryC1/StrS aminotransferase [Chlorobium limicola DSM 245]
	11	64	8,98E-11	1) DegT/DnrJ/EryC1/StrS aminotransferase [Cyanothece sp. PCC 8801] Glutamine--scyllo-inositol transaminase [Cyanothece sp. PCC 8802]
HOT:454	26	57	1,20E-25	1) aminotransferase DegT [Aminicenantes bacterium SCGC AAA252-F08]
	21	65	4,32E-23	1) perosamine synthase, partial [Escherichia coli TW14313]
MontereyBay	23	62	7,55E-24	1) DegT/DnrJ/EryC1/StrS aminotransferase [Thermoanaerobacterium saccharolyticum JW/SL-YS485]
	24	55	1,96E-23	1) truncated perosamine synthase [Vibrio cholerae O1 biovar El Tor]
N. Pac. Line67	43	41	4,14E-21	1) DegT/DnrJ/EryC1/StrS aminotransferase [Pseudoalteromonas sp. SM9913]
	22	44	1,18E-23	1) Select seq gb|ETR71599.1| perosamine synthetase [Candidatus Magnetoglobus multicellularis str. Araruama]
PBSM	80	33	9,25E-44	1) *glutamine--scyllo-inositol transaminase [Thioalkalivibrio sulfidophilus HL-EbGr7]*
AlvinellaPompejana	68	37	1,07E-38	1) *Glutamine--scyllo-inositol transaminase [Clostridium sp. DL-VIII]*
	50	44	3,55E-42	1) DegT/DnrJ/EryC1/StrS aminotransferase family protein [Desulfovibrio magneticus RS-1]
GuaymasBasin	48	47	4,76E-35	1) *Glutamine--scyllo-inositol transaminase [Methylobacter tundripaludum SV96]*
	10	68	1,61E-11	1) DegT/DnrH/EryC1/StrS aminotransferase
BotanyBay:Sanger	76	49	1,46E-69	1) hypothetical protein [Flavobacterium sp. MS220-5C]<br>2) aminotransferase DegT [gamma proteobacterium SCGC AAA001-B15]
Bacterioplankton	23	64	1,63E-27	1) perosamine synthase, partial [Escherichia coli TW09195]
BATS	23	64	7,21E-24	1) DegT/DnrH/EryC1/StrS aminotransferase family protein [alpha proteobacterium HIMB5]
BermudaOceanic	49	44	4,75E-40	1) perosamine synthetase, partial [Burkholderia pseudomallei 354a]
	24	74	1,00E-31	
CellCapture	30	71	2,14E-34	1) perosamine synthetase [Delftia acidovorans CCUG 274B]
Drifting-ESP	42	46	9,91E-36	1) *pleiotropic regulatory protein [Microscilla marina ATCC 23134]*
	25	74	9,61E-26	1) putative perosamine synthetase Per [Rubrivivax gelatinosus IL144]
HydrothermalVent	81	32	1,55E-39	1) aminotransferase, DegT/DnrJ/EryC1/StrS family [Arcobacter butzleri 7h1h]
	60	45	2,87E-49	1) *glutamine--scyllo-inositol transaminase [Clostridium botulinum B str. Osaka05]*
TermiteGut	70	44	3,77E-56	1) DegT/DnrJ/EryC1/StrS aminotransferase [Polaromonas naphthalenivorans CJ2]
	53	55	1,76E-57	1) *glutamine--scyllo-inositol transaminase [Azospirillum sp. CAG:260]*
GutlessWorm	85	36	4,33E-52	1) *putative pyridoxal phosphate-dependent enzyme [Legionella oakridgensis ATCC 33761 = DSM 21215]*
	67	42	2,70E-44	

Metagenome	Coverage (%)	Identity (%)*	E-value	Best matches of NCBI BLAST of metagenomic hits
HypersalineMat	72	37	3,05E-47	
	59	52	2,51E-58	1) perosamine synthetase [Mycobacterium intracellulare MOTT-64]
IceMetagenome	21	71	1,94E-24	1) putative perosamine synthetase Per [Rubrivivax gelatinosus IL144]
WesternChannelOMM	50	32	2,62E-08	1) *hypothetical protein [unclassified Aminicenantes]*<br>2) *DegT/DnrJ/EryC1/StrS aminotransferase [Caulobacter segnis ATCC 21756]*
	29	61	6,67E-32	1) perosamine synthetase [Escherichia coli DEC4E]
	13	76	1,22E-10	1) perosamine synthetase [Escherichia coli DEC4E]
WashintonLake	96	33	2,81E-45	
	58	63	2,31E-82	1) putative perosamine synthetase Per [Rubrivivax gelatinosus IL144]
MILOCO:454Shotgun	45	67	2,3746E-53	1) *glutamine--scyllo-inositol transaminase [Geobacter sp. M21]*
	11	83	3,8601E-58	1) perosamine synthetase, Per protein [Aeromonas hydrophila subsp. hydrophila ATCC 7966]
HOT:Sanger	90	48	3,20E-38	
	76	50	5,82E-77	1) cystathione beta-synthase modulated DegT/DnrJ/EryC1/StrS aminotransferase [Desulfobacula toluolica Tol2]
	10	68	4,38E-35	
YLAKE	47	57	6,88E-56	1) putative perosamine synthetase Per [Rubrivivax gelatinosus IL144]
	29	73	9,20E-33	
AntarcticaAquatic:454	43	61	2,34E-52	1) perosamine synthetase [Delftia acidovorans CCUG 274B]
	11	84	2,67E-25	
BotanyBay:454	44	53	1,31E-35	1) perosamine synthetase [Escherichia coli DEC7E]
	31	70	2,47E-31	
AntarcticaAquatic:Sanger	82	56	3,45E-79	1) DegT/DnrJ/EryC1/StrS family aminotransferase [Pseudomonas plecoglossicida NB2011]
	11	65	3,66E-11	1) perosamine synthetase [Methylibium petroleiphilum PM1]
BisonMetagenome	89	36	7,53E-53	
	77	45	3,45E-68	1) aminotransferase DegT [Calescamantes bacterium JGI 0000106-G12]
GOS	94	32	5,65E-40	
	71	60	2,61E-90	1) perosamine synthetase [Escherichia coli DEC7E]
	12	79	3,40E-30	
MILOCO:454	53	34	1,71E-24	1) DegT/DnrJ/EryC1/StrS aminotransferase [Paenibacillus sp. JDR-2]
	31	63	1,39E-35	1) perosamine synthetase [Delftia acidovorans CCUG 274B]
wbdI (149 aa)				
SargassoSea	36	46	3,27E-07	1) GDP-mannose mannosyl hydrolase [Gammaproteobacteria bacterium MOLA455]
MontereyBay transect	46	43	7,33E-10	1) Select seq ref|WP_006158751.1| GDP-mannose mannosyl hydrolase [Cupriavidus basilensis] >gb|EHP42139.1| NUDIX hydrolase [Cupriavidus basilensis OR16]
HOT:454	48	50	8,53E-11	1) hypothetical protein P9515_07251 [Prochlorococcus marinus str. MIT 9515]<br>2) GDP-mannose mannosyl hydrolase NudD [Escherichia] >gb|EOU42568.1| hypothetical protein WC5_03783 [Escherichia coli KTE114]
	28	61	2,03E-08	

Metagenome	Coverage (%)	Identity (%)*	E-value	Best matches of NCBI BLAST of metagenomic hits
MontereyBay	45	39	4,62E-09	1) GDP-mannose mannosyl hydrolase [Shewanella violacea DSS12] >ref|WP_013050747.1| GDP-mannose mannosyl hydrolase NudD [Shewanella violacea] >dbj|BAJ01439.1| GDP-mannose mannosyl hydrolase [Shewanella violacea DSS12]
N. Pac. Line67	60	44	3,26E-19	1) hypothetical protein [Pseudomonas psychrophila]<br>2) GDP-mannose mannosyl hydrolase [Escherichia coli KTE171]
SalternMetagenome	25	62	5,43E-07	1) GDP-mannose mannosyl hydrolase [Pseudomonas sp. TKP] >ref|WP_024074910.1| GDP-mannose mannosyl hydrolase [Pseudomonas sp. TKP] >gb|AHC35182.1| GDP-mannose mannosyl hydrolase [Pseudomonas sp. TKP]
GuaymasBasin	67	39	6,13E-15	1) GDP-mannose mannosyl hydrolase NudD [Pseudoalteromonas sp. NJ631]
Bacterioplankton	57	38	1,70E-09	1) GDP-mannose mannosyl hydrolase [Gammaproteobacteria bacterium MOLA455]
BATS	69	41	2,29E-13	1) NUDIX hydrolase [Glaciecola sp. HTCC2999]
Drifting-ESP	73	48	1,01E-25	1) Select seq ref|WP_010133600.1| GDP-mannose mannosyl hydrolase NudD [Microbulbifer agarilyticus]
HypersalineMat	97	47	7,95E-31	1) GDP-mannose mannosyl hydrolase [Escherichia coli KTE171]
WesternChannelOMM	89	34	2,62E-12	
	76	48	1,94E-27	1) NUDIX hydrolase [Escherichia coli DH1] >ref|WP_000971422.1| GDP-mannose mannosyl hydrolase NudD [Escherichia coli]
WashintonLake	98	46	4,83E-32	1) NUDIX hydrolase [Methylobacter tundripaludum SV96]
MILOCO:454Shotgun	98	46	1,26E-27	1) GDP-mannose mannosyl hydrolase [Sulfurovum sp. AR]
HOT:Sanger	99	45	2,12E-28	1) GDP-mannose mannosyl hydrolase NudD [Desulfurobacterium sp. TC5-1]
YLAKE	75	43	9,41E-19	1) GDP-mannose mannosyl hydrolase NudD [Photobacterium profundum] >gb|EAS44611.1| putative glycosyl transferase in colanic acid biosynthesis [Photobacterium profundum 3TCK]
AntarcticaAquatic:454	94	47	1,39E-26	1) GDP-mannose mannosyl hydrolase [Sulfurovum sp. AR]
	32	64	2,27E-26	
BotanyBay:454	62	41	5,02E-13	1) GDP-mannose mannosyl hydrolase [Gammaproteobacteria bacterium MOLA455]
AntarcticaAquatic:Sanger	100	46	6,56E-33	1) hypothetical protein O185_04020 [Photorhabdus temperata J3]<br>2) GDP-mannose mannosyl hydrolase NudD [Microbulbifer agarilyticus]
	32	64	1,13E-09	
BisonMetagenome	99	44	7,22E-31	1) GDP-mannose mannosyl hydrolase NudD [Pseudoalteromonas haloplanktis] >gb|EGI74685.1| GDP-mannose mannosyl hydrolase [Pseudoalteromonas haloplanktis ANT/505]
GOS	100	47	4,68E-32	1) GDP-mannose mannosyl hydrolase NudD [Escherichia coli] >gb|EGX07300.1| GDP-mannose mannosyl hydrolase [Escherichia coli G58-1] >gb|ESD68286.1| GDP-mannose mannosyl hydrolase [Escherichia coli 908541]
MILOCO:454	74	49	4,90E-19	1) GDP-mannose mannosyl hydrolase [Gammaproteobacteria bacterium MOLA455]
pqaB (548 aa)				
SalternMetagenome	6	73	3,87E-10	1) UDP phosphate-alpha-4-amino-4-deoxy-L-arabinose arabinosyl transferase [Serratia marcescens EGD-HP20]
BermudaOceanic	15	45	3,86E-13	1) undecaprenyl phosphate-alpha-4-amino-4-deoxy-L-arabinose arabinosyl transferase domain protein [Shigella flexneri 1235-66]
CellCapture	15	44	6,40E-11	1) 4-amino-4-deoxy-L-arabinose transferase [Pseudomonas extremaustralis]
GutlessWorm	56	31	2,14E-08	1) undecaprenyl-diphospho-4-amino-4-deoxy-L-arabinose--lipid A 4-amino-4-deoxy-L-arabinose transferase [Geobacter metallireducens GS-15]

Metagenome	Coverage (%)	Identity (%)*	E-value	Best matches of NCBI BLAST of metagenomic hits
				>ref|WP_004513034.1| glycosyl transferase [Geobacter metallireducens]
IceMetagenome	12	49	7,92E-09	1) Glycosyl transferase family 39 [beta proteobacterium CB]
WesternChannelOMM	23	60	1,79E-32	1) dolichyl-phosphate-mannose-protein mannosyltransferase, partial [uncultured bacterium Contig267]
	8	76	3,78E-38	
WashintonLake	42	32	1,92E-17	1) glycosyl transferase family protein [Geobacter lovleyi]
	10	60	1,25E-11	
MILOCO:454Shotgun	16	49	2,09E-13	1) undecaprenyl phosphate-alpha-L-Ara4N transferase [Agarivorans albus MKT 106]
HOT:Sanger	45	30	4,47E-11	1) dolichyl-phosphate-mannose-protein mannosyltransferase family protein [Thermodesulfovibrio yellowstonii DSM 11347]
YLAKE	27	41	3,45E-11	1) UDP phosphate-alpha-4-amino-4-deoxy-L-arabinose arabinosyl transferase [Erwinia toletana]
AntarcticaAquatic:454	28	44	2,71E-18	1) dolichyl-phosphate-mannose-protein mannosyltransferase [delta proteobacterium NaphS2]
	10	67	7,26E-14	
BotanyBay:454	28	74	1,28E-42	1) dolichyl-phosphate-mannose-mannosyltransferase family protein [Shigella flexneri K-404]
AntarcticaAquatic:Sanger	50	33	3,16E-11	1) 4-amino-4-deoxy-L-arabinose transferase [Shewanella sediminis HAW-EB3] >ref|WP_012141269.1| UDP phosphate-alpha-4-amino-4-deoxy-L-arabinose arabinosyl transferase [Shewanella sediminis]
BisonMetagenome	28	32	3,29E-10	1) glycosyl transferase [Persephonella sp. KM09-Lau-8]
GOS	68	77	3,73E-138	1) 4-amino-4-deoxy-L-arabinose transferase [Citrobacter] >gb|EJF21645.1| undecaprenyl phosphate-alpha-4-amino-4-deoxy-L-arabinose arabinosyl transferase [Citrobacter sp. A1]
MILOCO:454	30	41	5,12E-28	
	21	64	1,00E-39	1) undecaprenyl phosphate-alpha-4-amino-4-deoxy-L-arabinose arabinosyltransferase [Escherichia coli 1827-70]
yfbI (550 aa)				
MontereyBay	14	48	2,44E-09	1) glycosyl transferase family protein [Pirellula staleyi DSM 6068]
N. Pac. Line67	7	100	1,30E-12	1) undecaprenyl phosphate-alpha-4-amino-4-deoxy-L-arabinose arabinosyl transferase domain protein, partial [Shigella flexneri 1235-66]
PBSM	19	35	1,16E-07	1) glycosyl transferase family 39 [Desulfurobacterium thermolithotrophum]
SalternMetagenome	6	81	2,13E-11	1) UDP phosphate-alpha-4-amino-4-deoxy-L-arabinose arabinosyl transferase [Serratia marcescens] >gb|ERH67401.1| UDP phosphate-alpha-4-amino-4-deoxy-L-arabinose arabinosyl transferase [Serratia marcescens EGD-HP20]
AlvinellaPompejana	28	30	2,08E-12	1) dolichyl-phosphate-mannose-protein mannosyltransferase family protein [Alvinella pompejana epibiont 6C6]
BermudaOceanic	28	34	1,10E-15	1) PMT family glycosyltransferase, 4-amino-4-deoxy-L-arabinose transferase [Anaerobaculum mobile DSM 13181] >ref|WP_014807695.1| UDP phosphate-alpha-4-amino-4-deoxy-L-arabinose arabinosyltransferase [Anaerobaculum mobile] >gb|AFM22472.1| PMT family glycosyltransferase, 4-amino-4-deoxy-L-arabinose transferase [Anaerobaculum mobile DSM 13181]
CellCapture	14	54	4,63E-19	1) 4-amino-4-deoxy-L-arabinose transferase, partial [Pseudomonas sp. R81]
GutlessWorm	40	36	9,17E-13	1) glycosyl transferase [Geobacter pickeringii]<br>2) 4-amino-4-deoxy-L-arabinose lipid A transferase [Erwinia iniecta]
HypersalineMat	21	36	4,95E-11	1) putative glycosyltransferase [Candidatus Jettenia caeni]
IceMetagenome	13	44	5,12E-08	1) glycosyl transferase family protein [Delftia acidovorans SPH-1]

Metagenome	Coverage (%)	Identity (%)*	E-value	Best matches of NCBI BLAST of metagenomic hits
WesternChannelOMM	23	79	1,33E-48	1) dolichyl-phosphate-mannose-protein mannosyltransferase, partial [uncultured bacterium Contig267]
	18	100	1,62E-63	
WashintonLake	40	34	7,81E-21	1) glycosyl transferase family protein [Geobacter lovleyi]
	10	62	2,17E-12	
MILOCO:454Shotgun	17	44	3,93E-14	1) dolichyl-phosphate-mannose-protein mannosyltransferase [delta proteobacterium NaphS2]
HOT:Sanger	22	34	1,08E-09	1) 4-amino-4-deoxy-L-arabinose transferase and related glycosyltransferases of PMT family [Prochlorococcus marinus str. MIT 9301]
YLAKE	29	34	9,20E-12	1) glycosyl transferase family protein [Geobacter daltonii FRC-32]
	8	64	6,54E-07	
AntarcticaAquatic:454	25	52	1,75E-23	1) dolichyl-phosphate-mannose-protein mannosyltransferase [delta proteobacterium NaphS2]
	10	68	9,45E-13	
BotanyBay:454	28	99	1,54E-72	1) dolichyl-phosphate-mannose-protein mannosyltransferase [Escherichia coli] >gb|EFP99316.1| undecaprenyl phosphate-alpha-4-amino-4-deoxy-L-arabinose arabinosyltransferase [Escherichia coli 1827-70]
AntarcticaAquatic:Sanger	52	32	3,45E-19	1) glycosyltransferase [Marinobacter sp. ELB17]
BisonMetagenome	29	31	4,21E-09	1) hypothetical protein THERU_03255 [Thermocrinis ruber DSM 12173] 2) glycosyl transferase family 39 [Sulfurihydrogenibium sp. YO3AOP1]
GOS	68	73	1,57E-141	1) 4-amino-4-deoxy-L-arabinose transferase [Citrobacter] >gb|EJF21645.1| undecaprenyl phosphate-alpha-4-amino-4-deoxy-L-arabinose arabinosyl transferase [Citrobacter sp. A1]
	28	81	2,57E-60	
MILOCO:454	31	44	1,23E-33	
	26	78	1,00E-55	1) hypothetical protein [Escherichia coli 908525] 2) Polymyxin resistance protein ArnT, undecaprenyl phosphate-alpha-L-Ara4N transferase; Melittin resistance protein PqaB [Escherichia coli IS25] >emb|CDK78748.1| Polymyxin resistance protein ArnT, undecaprenyl phosphate-alpha-L-Ara4N transferase; Melittin resistance protein PqaB [Klebsiella pneumoniae IS22]
mecA (668 aa)				
DeepMed	29	33	1,78E-21	1) penicillin-binding protein 2 [Sulfurifustis variabilis]
Yellowstone	11	31	8,76E-08	1) penicillin-binding protein [Synechococcus sp. JA-3-3Ab]
AcidMine	40	31	8,17E-28	1) Peptidoglycan glycosyltransferase [Leptospirillum sp. Group II 'C75'] 2) penicillin-binding protein 2 [Leptospirillum sp. Group IV 'UBA BS']
MontereyBay transect	14	37	7,01E-08	1) penicillin-binding protein [Candidatus Pelagibacter ubique]
HOT:454	13	45	1,17E-10	1) penicillin-binding protein [Candidatus Pelagibacter ubique]
MontereyBay	13	40	2,28E-08	1) penicillin-binding protein 2 (PBP-2) [Leeuwenhoekiella blandensis MED217]
BotanyBay:Sanger	44	30	7,77E-17	1) penicillin-binding protein [Candidatus Pelagibacter ubique] >gb|EAS85327.1| Penicillin binding protein transpeptide [Candidatus Pelagibacter ubique HTCC1002]
AlvinellaPompejana	32	32	7,75E-18	1) Peptidoglycan glycosyltransferase [Parcubacteria group bacterium GW2011_GWA2_38_13] 2) penicillin-binding protein 2 [Dethiosulfatarculus sandiegensis]
GuaymasBasin	24	32	3,23E-14	1) penicillin-binding protein 2 [uncultured SUP05 cluster bacterium]
	11	44	7,04E-08	

Metagenome	Coverage (%)	Identity (%)*	E-value	Best matches of NCBI BLAST of metagenomic hits
Bacterioplankton	14	39	1,44E-09	1) penicillin-binding protein 2 [Methylohalobius crimeensis]
BATS	15	37	4,38E-11	1) penicillin-binding protein [Vibrio coralliilyticus OCN008]
BermudaOceanic	25	34	1,59E-16	1) penicillin-binding protein [Candidatus Pelagibacter ubique]
	14	43	1,48E-10	
CellCapture	11	41	8,33E-09	1) penicillin-binding protein 2 [Pseudomonas veronii]
Drifting-ESP	23	32	5,97E-13	1) hypothetical protein [alpha proteobacterium SCGC AAA015-O19]<br>2) penicillin-binding protein 2 [Rhodobacteraceae bacterium HIMB11]
HydrothermalVent	27	33	2,61E-14	1) penicillin-binding protein 2 [Desulfotomaculum alkaliphilum]
TermiteGut	41	31	1,89E-32	1) penicillin-binding protein 2 [Treponema primitia]
GutlessWorm	46	30	1,83E-28	1) penicillin-binding protein 2 [Desulfococcus multivorans DSM 2059]
HypersalineMat	38	32	1,21E-22	1) penicillin-binding protein 2 [bacterium L21-Spi-D4]
WesternChannelOMM	26	30	1,44E-12	1) penicillin-binding protein 2 [gamma proteobacterium HTCC2207]
	6	50	1,92E-09	
MILOCO:454Shotgun	25	37	3,56E-19	1) penicillin-binding protein 2 [marine gamma proteobacterium HTCC2148]
WashingtonLake	46	33	9,69E-33	1) penicillin-binding protein 2 [Syntrophorhabdus aromaticivorans]
YLAKE	25	36	2,67E-21	1) penicillin-binding protein 2 [Thermosediminibacter oceani DSM 16646]
	6	51	2,05E-10	
AntarcticaAquatic:454	24	37	1,33E-22	1) penicillin-binding protein 2 [Marinobacter algicola] >gb|EDM46594.1| Cell division protein FtsI/penicillin-binding protein 2 [Marinobacter algicola DG893]
	6	56	3,57E-10	
BotanyBay:454	25	33	1,35E-28	1) penicillin-binding protein 2 [Thiocapsa marina 5811]
	8	48	2,71E-12	
AntarcticaAquatic:Sanger	43	31	7,30E-26	1) penicillin binding protein transpeptide [Candidatus Pelagibacter ubique HTCC1062]
	6	48	4,74E-09	
BisonMetagenome	36	31	2,79E-23	1) peptidoglycan glycosyltransferase [Armatimonadetes bacterium DC]<br>2) penicillin-binding protein 2 [Geobacter sp. M18]
MILOCO:454	25	30	2,73E-11	1) penicillin-binding protein 2 [Pseudomonas sp. PAMC 25886]
GOS	55	34	3,25E-18	1) penicillin-binding protein, transpeptidase domain protein [SAR86 cluster bacterium SAR86E]
	7	44	3,20E-08	

* Only metagenomic hits having a protein sequence identity > 30% to known virulence genes are included. Note that hits with >30% but <31% identity are shown as 30% in the table.
