# Supplementary material for: Bacterial Human Virulence Genes across Diverse Habitats As Assessed by In silico Analysis of Environmental Metagenomes
Source: Front Microbiol. 2016 Nov 3;7:1712. doi: 10.3389/fmicb.2016.01712 (PMC5093120; doi:10.3389/fmicb.2016.01712)
Supplement: Supplementary file 4 [file Image1.PDF]

Supplementary Figure 1. Alignment of a single TBLASTN hit (Sbjct) of the WashingtonLake metagenome against the protein sequence of *wbdI* (A), and alignment of a single TBLASTN hit (Sbjct) of the GOS metagenome against the protein sequence of *pqaB* (B). Although the sequence identity of the environmental homologue to *wbdI* was relatively low (46%), the GDP-mannose binding site (grey shading) was present. In the case of *pqaB*, almost the entire sequence is conserved. Thus, the high identity (77%) indicate that the environmental homologue is functional.

A

```
WbdI   2    FITSDKFREIIKLVPLVSIDLLIENENGEYLFGLRNNRPAKNYFFVPGGRIRKNESIKNA  61
            FITSDKFR +++  PLVSIDLL++N  G+ L GLR NRPA+  +FVPGGR+ K+E++  A
Sbjct  1    FITSDKFRLVVESTPLVSIDLLLQNAAGQILLGLRRNRPAQGCWFVPGGRVLKDETLDAA  60

WbdI   62   FKRISSMELGKEYGISGSVFNGVWEHFYDDGFFSEGE---ATHYIVLCYTLKVLKSEL-N  117
            F+R++  ELG     S +   GV+EHFY D  F  GE    THY+VL Y LK+ + +L +
Sbjct  61   FRRLAHAELGTPLERSQARLLGVYEHFYSDSVFGSGETNPGTHYVVLGYQLKISEPDLQD  120

WbdI   118  LPDDQHREYLWLTKHQINAKQDVHNYSKNYF  148
            LP  QH +Y W +   + A   VH  ++ Y
Sbjct  121  LPQSQHEKYRWWSSTDMRASAMVHENTRAYL  151
```

Query coverage: 98%; Sequence identity: 46%

B

```
PqaB   9    LAFTAFIALYYVIPVNSRLLWQPDETRYAEISREMLASGDWIVPHFLGLRYFEKPIAGYW  68
            LA  AFIALY+++P+NS LLWQPDETRYAEISREML SGDWIVPHFLGLRYFEKPIAGYW
Sbjct  1    LACIAFIALYFILPINSPLLWQPDETRYAEISREMLTSGDWIVPHFLGLRYFEKPIAGYW  60

PqaB   69   INSLGQWLFGATNFGVRAGAILTTLLAAALVAWLTFRLWRDKRTALLASVIFLSLFAVYS  128
            +NS+GQWLFGA NFGVRAGAI  TLL A LVAWL  RLW+DKRTA+L+S IFL+ F+VYS
Sbjct  61   LNSIGQWLFGANNFGVRAGAIFATLLTALLVAWLAMRLWQDKRTAVLSSAIFLTFFSVYS  120

PqaB   129  IGTYAVLDPMIALWLTAGMCCFWQGMQATTRTGKIGMFLLLGATCGLGVLTKGFLALAVP  188
            IGTYAVLDP+IALWL AGMCCFW+GMQATTRT KI  FLLLG TCG+GV+TKGFLALAVP
Sbjct  121  IGTYAVLDPIIALWLMAGMCCFWEGMQATTRTRKIIAFLLLGLTCGMGVMTKGFLALAVP  180

PqaB   189  VVSVLPWVIVQKRWKDFLLYGWLAVLSCFVVVLPWAIAIARREADFWHYFFWVEHIQRFA  248
            V+SVLPWV  QKRWKD +LYG LAV+ C V+VLPW IAIARRE DFWHYFFWVEHIQRFA
Sbjct  181  VLSVLPWVFAQKRWKDLILYGLLAVIGCVVIVLPWGIAIARREPDFWHYFFWVEHIQRFA  240

PqaB   249  MSDAQHKAPFWYYLPVLLAGSLPWLGLLPGALKLGWRERN---GAFYLLGWTIMPLLFFS  305
              DAQHKAP WYY+PV +AG+LPWLGLLPGAL+ GWRER+    A YLL W +MP+LFFS
Sbjct  241  QDDAQHKAPIWYYIPVFVAGTLPWLGLLPGALRTGWRERDVPRSALYLLSWIVMPILFFS  300

PqaB   306  IAKGKLPTYVLSCFAPIAILMARFVLHNVKEGVAALRVNGGINLVFGIIGIVAAFVVSSW  365
            IAKGKLPTY+LSCFAP+AIL ARF L   ++G AALRVNG IN++FG+ GIVA  +VS+
Sbjct  301  IAKGKLPTYILSCFAPLAILTARFGLQAAEKGSAALRVNGWINVLFGVTGIVATLLVSTL  360

PqaB   366  GPLKSPVWTHIET  378
            GPLK+PVW HIET
Sbjct  361  GPLKTPVWVHIET  373
```

Query coverage: 66%; Sequence identity: 77%
