# Supplementary material for: Bacterial Human Virulence Genes across Diverse Habitats As Assessed by In silico Analysis of Environmental Metagenomes
Source: Front Microbiol. 2016 Nov 3;7:1712. doi: 10.3389/fmicb.2016.01712 (PMC5093120; doi:10.3389/fmicb.2016.01712)

Supplementary Figure 2. Minimum evolution distance trees (A) and maximum parsimony trees (B) of environmental sequences homologous to the protein sequence of *papH* and *hlyA*. Only environmental sequences with a coverage >70%/139 aa (PapA) and >15%/150 aa (HlyA) are included. Clinical sequences are shown in red boldface type. Bootstrap values above 50% are displayed.

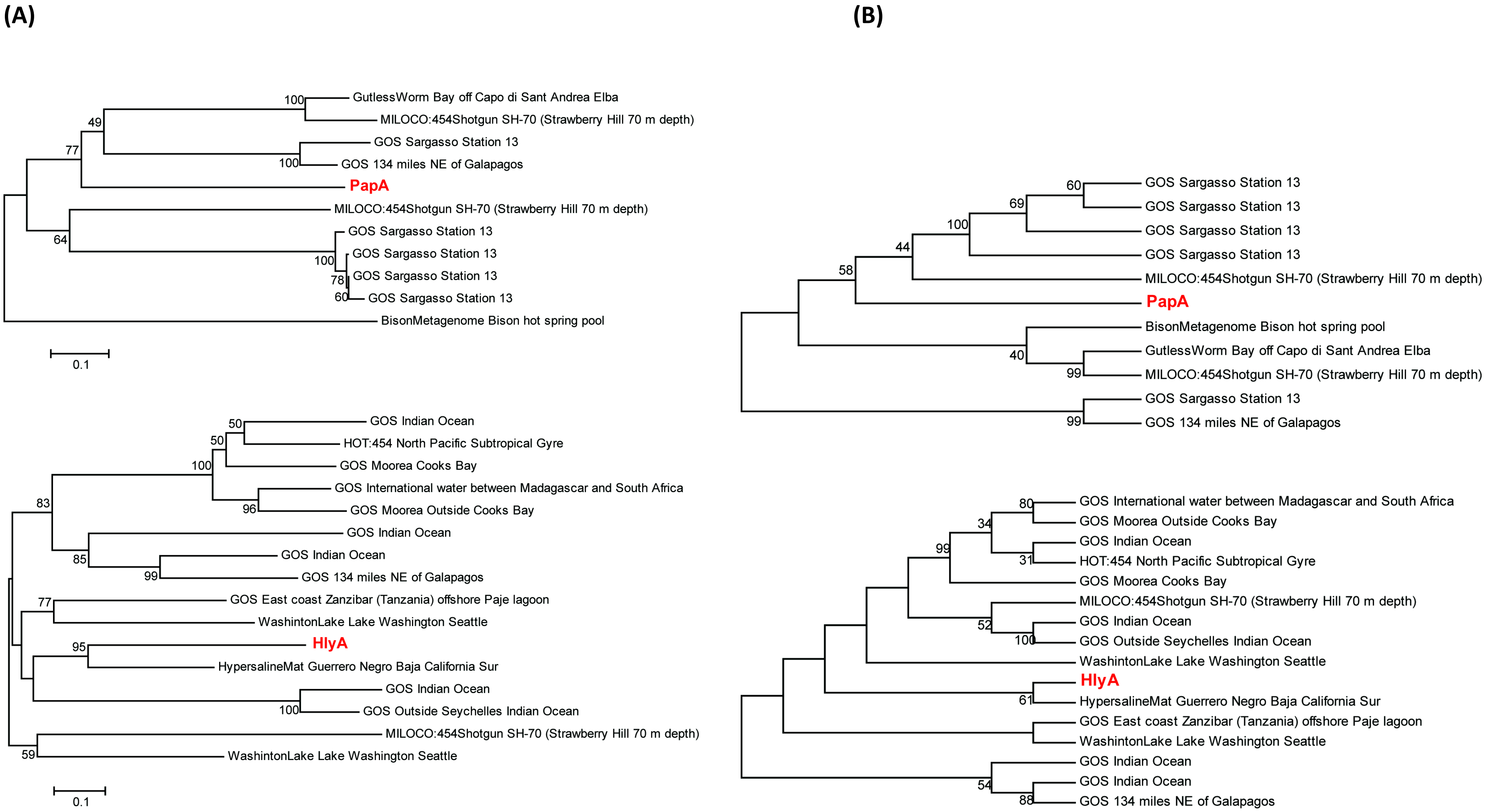

Supplement: Supplementary file 5 [file Image2.PDF]
